# Modulating Purothionin Accumulation and Signal Peptide Cleavage Fine‐Tunes Wheat Flour Gluten Properties to Improve Cookie‐Making Quality

**DOI:** 10.1002/advs.202512581

**Published:** 2026-01-07

**Authors:** Yijie Liu, Siyuan Chang, Zhaoheng Zhang, Tianqi Kang, Mingde Liu, Yuan Zong, Fei Ni, Yinguang Bao, Ruijie Zhang, Xiaobang Zhang, Jinkun Du, Mingming Xin, Zhaorong Hu, Jie Liu, Zhongfu Ni, Qixin Sun, Yingyin Yao

**Affiliations:** ^1^ State Key Laboratory of High‐Efficiency Production of Wheat‐Maize Double Cropping Frontiers Science Center for Molecular Design Breeding China Agricultural University Beijing China; ^2^ State Key Laboratory of Wheat Improvement College of Agronomy Shandong Agricultural University Taian Shandong China

**Keywords:** cookie, endoplasmic reticulum stress, low gluten, protein body, wheat

## Abstract

In contrast to many wheat (*Triticum aestivum*)‐based products that benefit from strong gluten development, cookies benefit from weaker gluten. However, the development of wheat varieties that produce flour optimal for cookie making remains limited. In this study, we identified the wheat mutant *low gluten protein 2* (*lgp2*), with reduced gluten content and a weakened gluten network, that significantly improved several aspects of cookie‐making performance. The *lgp2* phenotype is caused by a missense mutation in *LGP2* that affects the signal peptide cleavage site of the encoded protein. Map‐based cloning reveals that *LGP2* encodes alpha‐2‐purothionin, a member of the thionin family of small proteins with potential antimicrobial activity. The *lgp2* mutation leads to endoplasmic reticulum stress, abnormal protein body formation, and disrupted gluten development. Additionally, alpha‐2‐purothionin interacts with key seed storage proteins, contributing to gluten formation. Knockdown and overexpression studies confirmed that LGP2 affects gluten quantity and quality. Based on these findings, we propose dual genetic strategies targeting signal peptide processing and modulating *LGP2* expression to fine‐tune gluten properties for improved cookie quality. The *lgp2* allele offers great potential for breeding low‐gluten wheat varieties tailored for the production of cookies and other specialty food products.

## Graphical abstract

Dual genetic strategies for improving wheat processing quality by regulating purothionin accumulation to modulate gluten quantity and quality. The first strategy involves targeting signal peptide (SP) cleavage sites (e.g., through mutation) to indirectly reduce gluten content, thereby disrupting gluten network formation. The second strategy focuses on modulating purothionin gene expression (e.g., via knockdown or knockout) to fine‐tune both the quantity and quality of gluten.

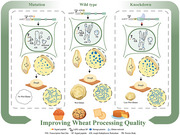

## 1 Introduction

Wheat (*Triticum aestivum* L.) is widely cultivated worldwide, serving as a staple food for millions of people [1, 2]. Cookies are a popular wheat flour–based product, representing versatile snacks in the bakery industry owing to their long shelf life, varied flavors and ingredients, and appealing textures [3]. The global cookie market was valued at approximately US$104.32 billion in 2023 and is projected to grow to US$167.69 billion by 2032 [4]. Wheat flour is a key ingredient in cookies and significantly affects the quality of the final product; therefore, developing new wheat varieties that produce flour with properties amenable to making cookies is crucial for meeting the growing demand from cookie producers.

Wheat varieties that are ideal for cookie making typically possess certain characteristics, including a soft kernel texture, weak gluten, and low protein content [5]. Compared with a hard kernel structure, a soft kernel structure produces smaller particles with less damaged starch during milling, leading to lower water absorption capacity [6]. Cookie dough requires flour with a low water absorption capacity to ensure that the dough remains hard and maintains good shaping properties, making it easy to handle and mold during production [7]. Excessive water absorption by flour from hard wheat can make the dough too soft to shape, leading to irregular cookie shapes during baking [8].

Low protein levels and low gluten strength are considered to be best for cookie production. The gluten network forms through the hydration and mixing of seed storage proteins, including glutenins and gliadins. This structure is built via disulfide bonds, which provide stability to the dough [9, 10]. For cookie production, it is important to have weak gluten, as excessive gluten development can lead to hard‐textured cookies. Moreover, gluten inhibits cookie spread during baking [11]. Therefore, cookie flour typically has a low protein content of approximately 7%–9%, which helps minimize gluten formation and achieve the desired texture. Gluten can be measured using the wet gluten content, which evaluates the gluten remaining after other dough components, such as starches, are washed away. A wet gluten content of less than 25% is required for cookie production (LS/T 3248‐2017); however, the wet gluten content of many soft wheat varieties exceed 26% (Figure S1A). This discrepancy highlights the need to reduce the wet gluten content and gluten strength of soft wheat to meet the quality standards for cookie flour, contributing to improved cookie‐making performance.

Researchers have focused on modifying gluten proteins to enhance cookie quality. Gluten is made up of high‐molecular‐weight glutenin subunits (HMW‐GSs), low‐molecular‐weight glutenin subunits (LMW‐GSs), and gliadins [12]. The ideal profile of HMW‐GSs for cookie production includes subunits 1Ax2^*^, 1Bx7, and 1Dx2+1Dy12 [13]. Knocking out the HMW‐GS *1Dy12* gene decreases gluten strength while increasing cookie diameter without affecting grain yield [14]. Introducing exogenous gliadins from *Psathyrostachys huashanica*, a wheat relative, reduces the thickness of cookies (termed biscuits in British English) but significantly increases their diameter and spread rate [15]. Mutating the γ‐gliadin gene *LGP1* (*Low gluten protein* 1) at the signal peptide cleavage site triggers endoplasmic reticulum (ER) stress and hinders the transport of seed storage proteins, ultimately reducing gluten protein content [16]. However, progress in the genetic improvement of wheat varieties intended for cookie production has been slow owing to a lack of key genes suitable for breeding.

Here, we identified the ethyl methanesulfonate (EMS)‐induced wheat mutant *lgp2* (*low gluten protein 2*), which exhibits lower gluten protein content, weakened gluten network strength, and enhanced cookie‐making performance compared with wild‐type “Lumai 15.” This phenotype is attributed to a missense mutation at the signal peptide (SP) cleavage site of alpha‐2‐purothionin, which leads to ER stress, abnormal protein body (PB) formation, and a weak gluten network. We determined that alpha‐2‐purothionin functions in gluten formation by interacting with seed storage proteins; the knockdown of its encoding gene led to lower gluten and protein content. These findings establish dual genetic strategies—targeting SP cleavage sites and modulating purothionin gene expression (for fine‐tuning gluten quantity and quality), offering a promising approach for breeding speciality wheat varieties optimized for low‐gluten food applications.

## 2 Results

### 2.1 The *lgp2* Mutant Shows Weakened Gluten

**FIGURE 1:**
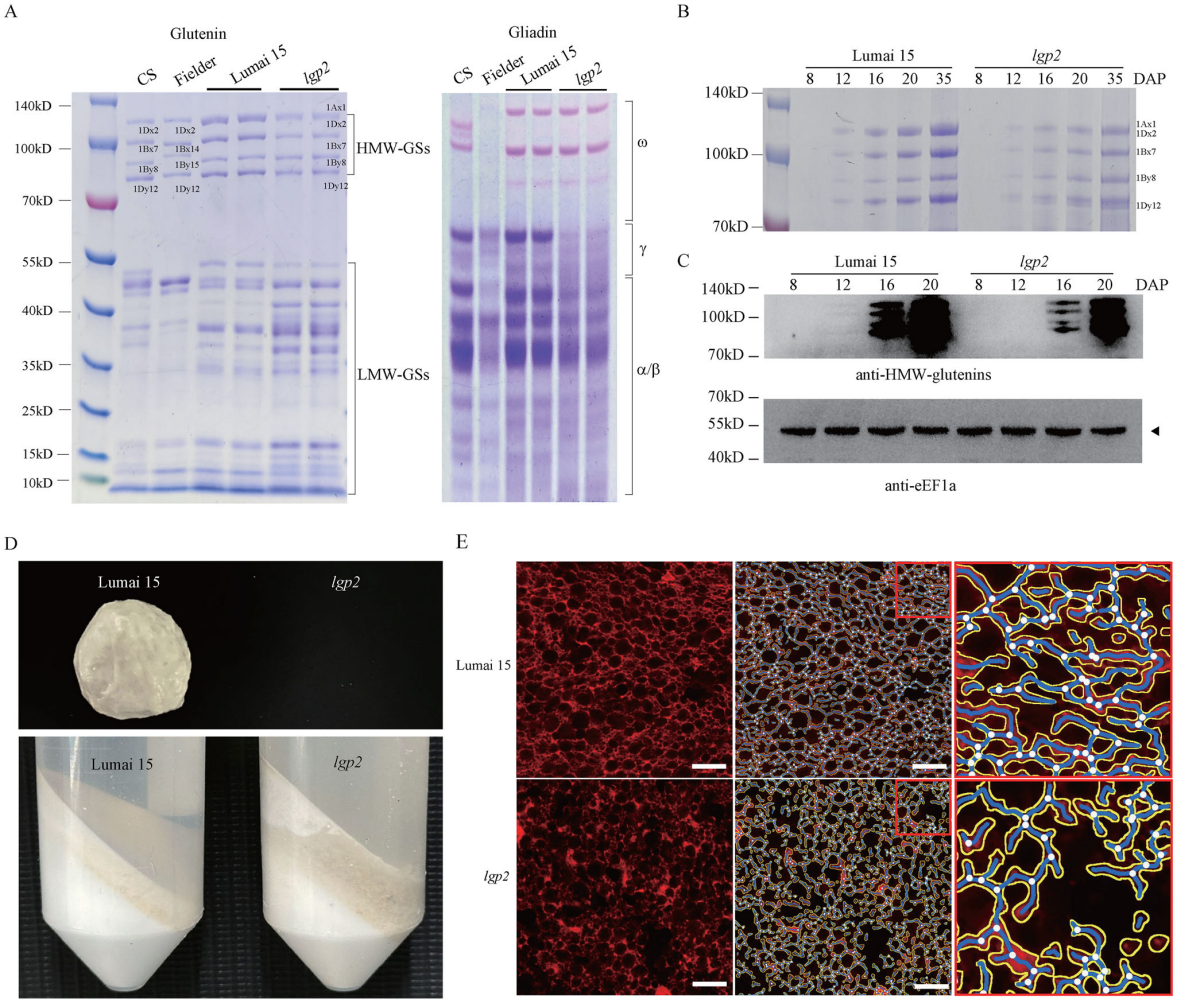
Characterization of the *lgp2* mutant. (A) Glutenin (left) and gliadin (right) profiles of Lumai 15 (WT) and *lgp2* grains visualized by Coomassie Blue staining of SDS‐PAGE gels and A‐PAGE gels. 1Ax1, 1Bx7 +1By8, and 1Dx2 + 1Dy12 presented the high molecular weight glutenin subunits (HMW‐GSs) on chromosome 1A, 1B, and 1D, respectively. Chinese Spring (CS) and Fielder are used as control varieties, with their high molecular weight glutenin subunit compositions being null, 1Bx7 +1By8, 1Dx2 + 1Dy12, and null, 1Bx14 +1By15, 1Dx2 + 1Dy12, respectively. (B) HMW‐GS level in Lumai 15 and *lgp2* grains harvested at different developmental stages visualized by Coomassie Blue staining of SDS‐PAGE gels. DAP, days after pollination. (C) Immunoblotting analysis of protein extracts prepared from Lumai 15 and *lgp2* grains harvested at different developmental stages. (D) Representative photographs of washed‐out gluten from flour (upper panel) and sediment from washed flour after centrifugation (lower panel) derived from Lumai 15 or *lgp2* grains. (E) Representative photographs of gluten network structure in Lumai 15 (upper panels) and *lgp2* (lower panels) dough samples stained with rhodamine. Yellow lines, blue lines, and white points represent the boundary, skeleton, and junction points of the gluten network, respectively. Scale bars: 50 µm.

The hexaploid wheat cultivar Lumai 15 is classified as soft wheat. However, Lumai 15 has a high gluten content of 33.2% in various environments (Figure S1B), which limits its potential as a high‐quality variety for cookie production. To address this issue, we created a mutant population derived from Lumai 15 using EMS and screened for mutants with reduced gluten protein levels. We identified the *low gluten protein 2* (*lgp2*) mutant, whose mature grains exhibit lower levels of HMW‐GS and gliadin but higher levels of LMW‐GS than Lumai 15 (Figure 1A; Figure S2A–D). In Lumai 15 seeds, HMW‐GSs begin to accumulate at 8 DAP, while HMW‐GS levels remain relatively low in *lgp2* seeds during the same developmental stages (Figure 1B,C). Gluten could not be separated from *lgp2* dough; instead, following the centrifugation of well‐mixed flour‐water suspensions from Lumai 15 and *lgp2*, a substance similar to gluten accumulated on the surface of the starch solution from the mutant (Figure 1D), suggesting that the integrity of the gluten network was weakened in the mutant.

To explore the structure of the gluten network in dough at the microscopic level, we examined Lumai 15 and *lgp2* dough samples using confocal laser scanning microscopy (Figure 1E). Compared with Lumai 15 dough, *lgp2* dough exhibited significantly less protein area (red in the left panel, outlined in yellow in the middle and right panels) and greater aperture area (black). These observations indicate that the gluten network is less connected in *lgp2* than in Lumai 15, with more gaps and irregularities. Furthermore, the fewer junction points (white dots in the middle and right panels) in *lgp2* reflect weaker cohesion within the gluten network (Figure 1E). Consequently, the *lgp2* mutant possessed a weak gluten network that could not be separated from the dough, in contrast to that of Lumai 15.

### 2.2 The *lgp2* Phenotype is Associated with a Missense Mutation in an Alpha‐2‐Purothionin Gene

**FIGURE 2:**
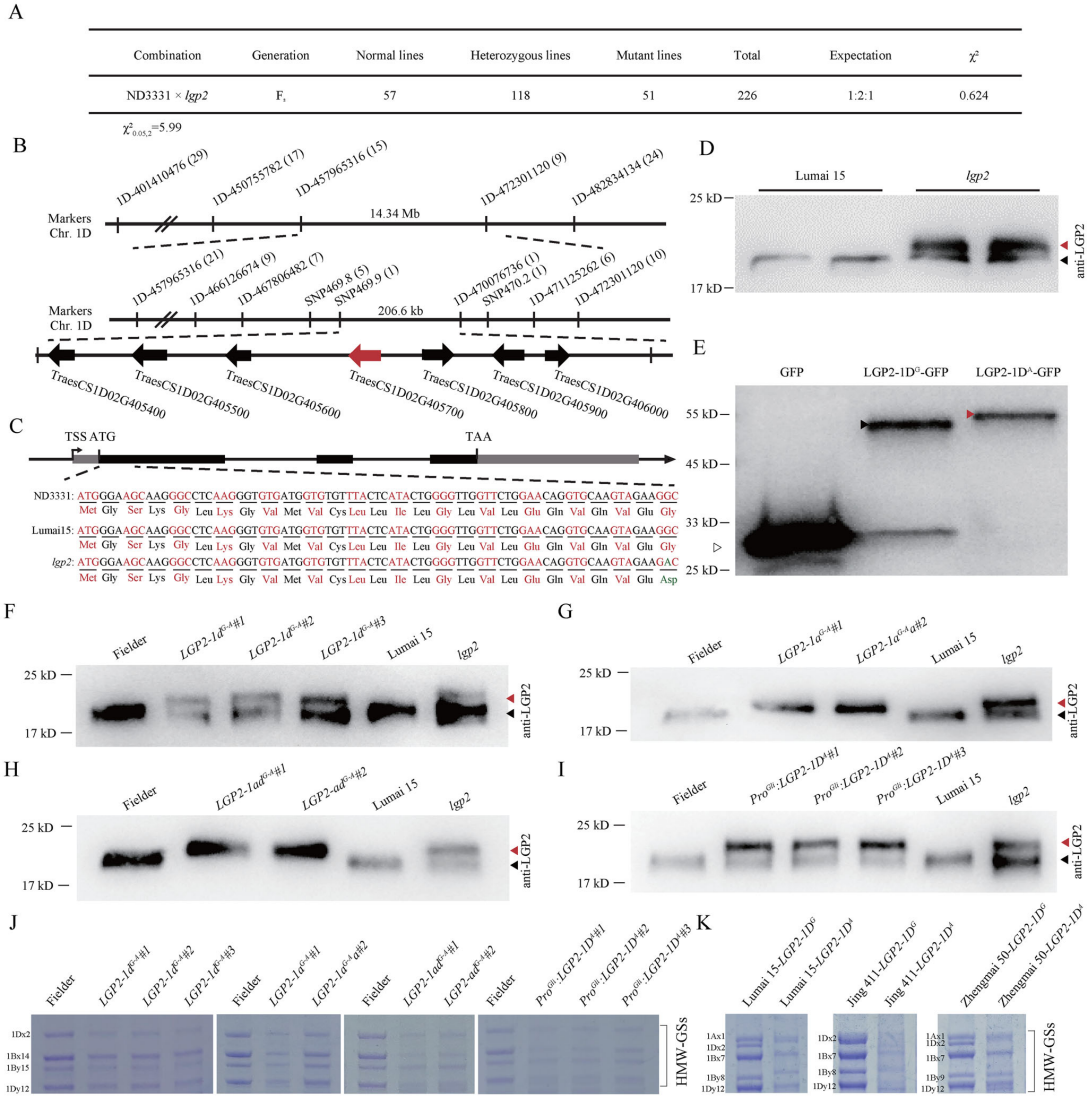
Map‐based cloning of the gene associated with the *lgp2* mutation. (A) Segregation ratio of F_2_ progeny derived from self‐pollination of ND3331 × *lgp2* F_1_ plants. The F_2_ plants were genotyped based on F_3_ seed phenotypes. (B) Fine mapping of the *LGP2* locus. The *LGP2* locus was narrowed to a 206.6‐kb physical region between markers *SNP469.9* and *ID‐470076736* on wheat chromosome 1D. This region includes seven predicted genes, among which TraesCS1D02G405700 is the candidate gene *LGP2* (red arrow). Molecular markers and the number of recombinants is shown. (C) Gene structure and mutation site of *LGP2*. Gray boxes, 5′ UTR or 3′ UTR. Black boxes, Exons. Lines, introns. (D) Immunoblot analysis of Lumai 15 (WT) and *lgp2*. Red arrowhead, mutant LGP2 protein. Black arrowhead, the proteins from the two other homoeologous genes of *LGP2*. (E) Immunoblot analysis of LGP2^G^‐GFP (from Lumai 15) and LGP2^A^‐GFP (from *lgp2*). Total proteins were extracted from wheat leaf protoplasts and probed with anti‐GFP antibodies. Black or red arrowhead, LGP2. White arrowhead, GFP. (F–I) Immunoblot analysis of gene‐edited lines (F–H) and overexpression lines (I). Black arrowheads, wild‐type LGP2; red arrowheads, mutant LGP2. (J) Comparison of glutenin in Fielder (control) and *lgp2* transgenic plants detected by SDS‐PAGE followed by Coomassie Blue staining. Glutenin proteins from one dry seed per line are shown. 1Ax1, 1Bx7 +1By8, and 1Dx2 + 1Dy12 presented the high molecular weight glutenin subunits (HMW‐GSs) composition is 1Ax1, 1Bx7 +1By8, and 1Dx2 + 1Dy12 on chromosomes 1A, 1B, and 1D, respectively. (K) Comparison of glutenin in backcrossed lines with the Lumai 15, Jing 411, and Zhengmai 50 genetic backgrounds containing *LGP2^G^* or *LGP2^A^* as detected by SDS‐PAGE followed by Coomassie Blue staining. One dry seed was examined from each line. HMW‐GS compositions: Lumai 15 (1Ax1,1Bx7+1By8 and 1Dx2+1Dy12); Jing 411 (Null, 1Bx7+1By8 and 1Dx2+1Dy12); Zhengmai 50 (1Ax1, 1Bx7+1By9 and 1Dx2+1Dy12).

To investigate the genetic inheritance and underlying cause of the *lgp2* trait, we constructed an F_2_ population containing 226 individuals derived from a cross between *lgp2* and Nongda 3331 (ND3331), a cultivar with a high level of HWM‐GS comparable with that of Lumai 15. We used HMW‐glutenin content as a marker for phenotyping. Seeds from 51 F_2_ individuals showed low HMW‐GS levels similar to those of *lgp2*, whereas seeds from 57 F_2_ individuals showed normal HMW‐GS levels similar to those of ND3331. Seeds from the remaining 118 individuals segregated for HMW‐GS content, indicating that those plants were heterozygous. These results are in agreement with a 1:2:1 segregation ratio (χ2 = 0.62< χ^2^_(0.05,2)_ = 5.99) (Figure 2A; Figure S3), suggesting that the *lgp2* mutant phenotype is associated with a single‐locus mutation.

We subjected genomic DNA from the *lgp2* mutant and ND3331 to whole‐genome resequencing. We developed markers based on the polymorphisms between these two genomes for gene mapping. We created 216 insertion–deletion (InDel) markers, which were evenly distributed across the 21 chromosomes. Notably, marker *1D‐457965316*, located on the long arm of chromosome 1D, was linked to the *lgp2* phenotype, as determined using 51 homozygous F_2_ plants that exhibited low HMW glutenin levels (χ2 = 56.53 > χ^2^_(0.05,2)_ = 5.99). We developed several markers (*1D‐401410476*, *1D‐450755782*, *1D‐472301120*, and *1D‐482834134*) on both sides of marker *1D‐457965316* and mapped the gene to within the interval between markers *1D‐457965316* and *1D‐472301120*, with a physical distance of 14.34 Mb. Using a larger F_2_ population of 1936 plants from the cross *lgp2* × ND3331, after developing seven additional markers (*1D‐466126674*, *1D‐467806482*, *SNP469.8*, *SNP469.9*, *1D‐470076736*, *SNP470.2*, and *1D‐471125262*), the candidate gene was narrowed down to a 206.6‐kb interval between markers *SNP469.9* and *1D‐470076736*.

Within this region, we identified seven annotated high‐confidence genes based on the Chinese Spring IWGSC RefSeq v1.1 reference genome (Figure 2B). We sequenced the region from TSS to TES, including all exons/introns and UTRs, and promoter (2 kb upstream of the transcription start site) of these seven genes in *lgp2*, Lumai 15, and ND3331. Only one G‐to‐A transition within the coding region of TraesCS1D02G405700 was detected in *lgp2* compared with Lumai 15 and ND3331 (Figure 2C). We also compared the expression levels of these seven genes between *lgp2* and Lumai 15 by RNA‐seq using endosperm from seeds at 15 and 20 days after pollination (DAP). None of them showed significant changes in expression in the mutant [log_2_(fold change) ≥0.8 and false discovery rate (FDR) < 0.05] (Tables S1 and S2), compared to Lumai 15. These findings indicate that TraesCS1D02G405700 is the candidate gene associated with the *lgp2* phenotype. We therefore named this gene *LGP2*. *LGP2* encodes an alpha‐2‐purothionin protein, a member of the thionin family of small proteins with potential antimicrobial activity. A G‐to‐A transition in the nucleotide sequence causes an amino acid change from Gly (glycine, GGC) to Asp (aspartic acid, GAC) at position 27, located at the end of the SP (Figure 2C).

### 2.3 An Unprocessed Signal Peptide in Alpha‐2‐Purothionin Results in the *lgp2* Phenotype

The SP of a protein, which contains a signal peptidase cleavage site at its C terminus, is crucial for protein processing and secretion [9, 17]. Cleavage of the SP converts the protein into its mature form, enabling further modifications and proper folding [9]. Studies in barley and wheat have shown that thionins in the endosperm are synthesized as preproproteins containing an endoplasmic reticulum (ER)‐targeting signal peptide. To confirm that the G‐to‐A mutation of *LGP2* leads to failed SP cleavage, we generated an antibody against LGP2 and measured its levels in *lgp2* and Lumai 15 by immunoblotting. The Lumai 15 sample showed a single band, representing the total abundance of three proteins encoded by genes from chromosomes 1A, 1B, and 1D, which share high sequence similarity. By contrast, the *lgp2* mutant exhibited an additional band with a molecular weight approximately 2–3 kD greater than that of the corresponding band in Lumai 15 (Figure 2D). This finding indicates the presence of mutant LGP2 protein from chromosome 1D (Figure 2D). We transiently transformed wheat leaf protoplasts with GFP‐tagged full‐length LGP2 from Lumai 15 (*LGP2‐1D^G^*) or *lgp2* (*LGP2‐1D^A^*) and examined the sizes of the resulting tagged proteins by immunoblotting with anti‐GFP antibody. LGP2‐1D^A^‐GFP was approximately 2–3 kD larger than LGP2‐1D^G^‐GFP (Figure 2E). These results indicate that the G‐to‐A substitution in the *lgp2* mutant causes a failure of SP cleavage.

To confirm the functional effect of the G‐to‐A mutation in *LGP2*, we used prime editing to edit the *LGP2‐1D* gene from *LGP2‐1D^G^* to *LGP2‐1D^A^* in wheat cultivar Fielder, which contains the *LGP2‐1D^G^* gene and shows normal gluten levels [18]. We designed the spacer sequence for spCas9 and the primer binding site of the prime‐editing guide RNA (pegRNA) to target both *LGP2‐1A* and *LGP2‐1D*; *LGP2‐1B* was not targeted owing to the presence of a single‐nucleotide polymorphism (SNP) in the primer binding site (Figure S4A). Among the seven independent transgenic lines generated, three lines (*LGP2‐1d^G‐A^#1/#2/#3*) contained edited *LGP2‐1D* alone, where G at the 80th nucleotide was edited to A; two lines (*LGP2‐1a^G‐A^#1/#2*) contained edited *LGP2‐1A* alone; and two lines (*LGP2‐1ad^G‐A^#1/#2*) contained both edited *LGP2‐1D* and edited *LGP2‐1A* (Figure S4B). In the immunoblot analysis, all these lines exhibited larger bands, representing mutated LGP2 protein (red arrowheads), than Fielder (Figure 2F–H). We also generated lines harboring the *LGP2‐1D^A^* coding sequence containing the G‐to‐A mutation driven by the endosperm‐specific promoter of the γ‐gliadin gene (*Pro^Gli^*) in the Fielder background via transformation. Three transgenic lines (*Pro^Gli^:LGP2‐1D^A^#1*/*#2/#3*) produced two bands representing the mutated (red arrowhead) and normal (black arrowhead) LGP2 proteins in the immunoblot analysis (Figure 2I).

The genome‐edited lines *LGP2‐1d^G‐A^*, *LGP2‐1a^G‐A^*, and *LGP2‐1ad^G‐A^*, along with the transgenic *Pro^Gli^:LGP2‐1D^A^* lines, showed similar phenotypes to *lgp2*, with lower levels of HMW‐GS than Fielder (Figure 2J). To assess the potential effects of the G‐to‐A mutation in *LGP2* in different backgrounds, we crossed the *lgp2* line with two additional soft wheat cultivars: Jing 411 and Zhengmai 50. In the backcross generation, the lines containing the G‐to‐A mutation in *LGP2* exhibited lower glutenin levels than those without the mutation (Figure 2K). These results indicate that the G‐to‐A mutation in *LGP2* is sufficient for generating the phenotypic alteration observed in *lgp2* seeds.

### 2.4 *lgp2* Exhibits ER Stress and Abnormal Protein Bodies

**FIGURE 3:**
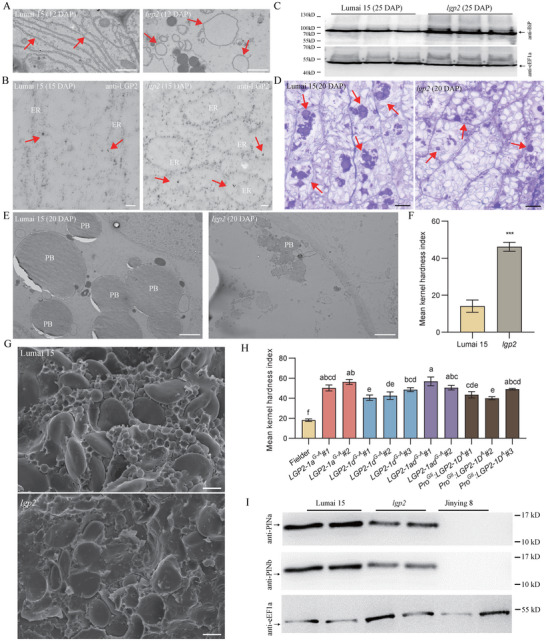
*lgp2* exhibits ER stress, abnormal protein bodies, altered puroindoline accumulation, and hard grain texture. (A) Representative TEM images of the ER in 12‐DAP starchy endosperm of Lumai 15 (left) and *lgp2* (right) processed by conventional chemical fixation. Red arrows indicate normal ER or abnormal ER. Scale bars: 2 µm. (B) Representative TEM micrographs showing immunogold labeling using anti‐LGP2 antibody in 15‐DAP Lumai 15 (left) and *lgp2* (right) starchy endosperm cells. Samples of starchy endosperm were processed by high‐pressure freezing (HPF). ER, endoplasmic reticulum. Red arrows indicate gold particles. Scale bars: 100nm. (C) Immunoblot analysis of the ER molecular chaperone BiP in 25‐DAP endosperm of Lumai 15 and *lgp2*. (D) Representative light micrographs of crystal violet–stained transverse sections of the middle regions of Lumai 15 and *lgp2* seeds at 20 DAP. Red arrows indicate protein bodies. Scale bars: 50 µm. (E) Representative TEM images of protein bodies (PB) in 20‐DAP starchy endosperm of Lumai 15 (left) and *lgp2* (right). Scale bars: 2 µm. (F) Comparison of grain hardness in Lumai 15 and the *lgp2* mutant measured using the Perten Single Kernel Characterization System (SKCS) 4100. Data are means ± s.d. (*n* = 6 biologically independent samples). (G) SEM analysis of Lumai 15 and *lgp2* endosperm. Scale bars: 10 µm. (H) Comparison of grain hardness in Fielder (control) and *lgp2* transgenic plants. Data are means ± s.d. (*n* = 3 biologically independent samples). (I) Immunoblot analysis of the hardness‐related proteins PINa and PINb in mature Lumai 15, *lgp2*, and Jinying 8 (AABB genome) grains. In (F), *P* values are from two‐sided Student's *t*‐tests (^***^p  < 0.001). In (H), Group differences were assessed by Welch's ANOVA followed by Games–Howell post‐hoc tests. Different letters indicate significant differences at *p* <0.05. Source data are provided as a Source Data file.

The precursor of purothionin is biosynthesized by membrane‐bound polysomes and is likely directed into the ER by its signal peptide [19]. This suggests that the unprocessed purothionin, resulting from a failure to cleave the signal peptide in endosperm cells, may trigger an ER stress response. We analyzed the ultrastructures of Lumai 15 and *lgp2* endosperm cells at 12 days after pollination (DAP) by conventional chemical fixation transmission electron microscopy (TEM) and noticed distinct ER morphologies between Lumai 15 and *lgp2* (Figure 3A). Lumai 15 endosperm cells showed a continuous, sheet‐like ER structure, whereas *lgp2* endosperm contained an abnormal ER structure, including ring‐like and swollen rough ER (Figure 3A). To confirm the localization of LGP2 to the ER, we performed immunoelectron microscopy on high‐pressure frozen 15‐DAP endosperm samples from Lumai 15 and *lgp2* grains using an anti‐LGP2 antibody. Gold particles were observed in the ER lumen in both Lumai 15 and *lgp2* (Figure 3B), indicating that LGP2 localizes to the ER. We transiently co‐expressed constructs harboring LGP2^G^‐GFP (Lumai 15) or LGP2^A^‐GFP (*lgp2*) with the ER marker RFP‐HDEL in wheat protoplasts. Both LGP2^G^‐GFP and LGP2^A^‐GFP localized to the ER, forming aggregates around the nuclei and plasmalemma (Figure S5).

Increased accumulation of the chaperone Immunoglobulin Binding Protein (BiP) is an important feature of ER stress [20]. The BiP content was substantially higher in 25‐DAP endosperm of *lgp2* than in that of Lumai 15 (Figure 3C), implying that ER stress was triggered in the mutant. Moreover, transcriptome analysis (Table S3) showed that 11 ER‐stress‐associated genes were expressed at a higher level in *lgp2* than in Lumai 15, including the plant‐specific ER stress–related transcription factor gene *BZIP50* [21], *ER‐LOCALIZED DNAJ FAMILY 3A* (*ERDJ3A*) [22], *ER LUMINAL‐BINDING PROTEIN 4* (*BIP4*) [23], and genes encoding proteins in the ER‐associated protein degradation (ERAD) pathway [24], including *PROTEIN DISULFIDE ISOMERASE* (Figure S6). Altogether, these results suggest that ER stress is substantially triggered in *lgp2*, likely owing to the accumulation of unprocessed purothionin in the ER.

In semi‐thin sections of *lgp2* seeds at 20 DAP, protein bodies were considerably smaller than those in Lumai 15 (Figure 3D). To further investigate these changes at higher resolution, we performed TEM on samples subjected to high‐pressure freezing. Unlike the uniformly distributed circular protein bodies in Lumai 15, the protein bodies in *lgp2* exhibited an irregular shape and a substantially reduced size (Figure 3E). Additionally, transcriptome analysis at 15 and 20 DAP indicated that the expression levels of major storage proteins, including glutenins and gliadins, were not significantly reduced in *lgp2* (Tables S7 and S8). These results reveal that seed storage protein accumulation is disrupted in the *lgp2* mutant, which is independent of their transcription.

### 2.5 The *lgp2* Mutant Shows Hard Grain Texture

Mature *lgp2* grains exhibited greater hardness than those of Lumai 15 (Figure 3F). In *lgp2*, the starch granules were compact and tightly integrated with the protein matrix, whereas in Lumai 15, the starch granules were loosely packed and only weakly adhered to the protein matrix (Figure 3G). The genome‐edited lines *LGP2‐1a^G‐A^*, *LGP2‐1d^G‐A^*, and *LGP2‐1ad^G‐A^*, along with the transgenic *Pro^Gli^:LGP2‐1D^A^* lines, also exhibited greater kernel hardness than Lumai 15 (Figure 3H).

The hardness of hexaploid wheat grains is primarily determined by two proteins encoded by genes on chromosome 5D: puroindoline a (PINa) and puroindoline b (PINb) [25]. Lumai 15 PINa and PINb proteins contribute to a soft kernel phenotype, whereas mutations in either *PINa* or *PINb* that result in changes to the amino acid sequence are associated with hard kernels [26, 27, 28]. Consequently, we measured PINa and PINb levels in grains using specific antibodies. Levels were lower in *lgp2* grains than in those of Lumai 15, which corresponded to a greater kernel hardness (Figure 3I). RNA‐seq analysis revealed no differences in *PINa* and *PINb* expression in grains at 15 and 20 DAP (Figure S7), indicating that PINa and PINb proteins might be affected by ER‐stress associated degradation. Jinying 8, a tetraploid wheat variety without the D subgenome (AABB), was used as a negative control because it does not contain *PINa* or *PINb*. In addition, both PINa and PINb co‐localized with the ER marker RFP‐HDEL in transiently transformed wheat protoplasts, indicating that they localize to the ER (Figure S7). Therefore, the ER stress triggered by the mutation in *lgp2* might lead to lower levels of PIN, as well as of glutenins and gliadins.

### 2.6 LGP2 is Involved in Gluten Formation

**FIGURE 4:**
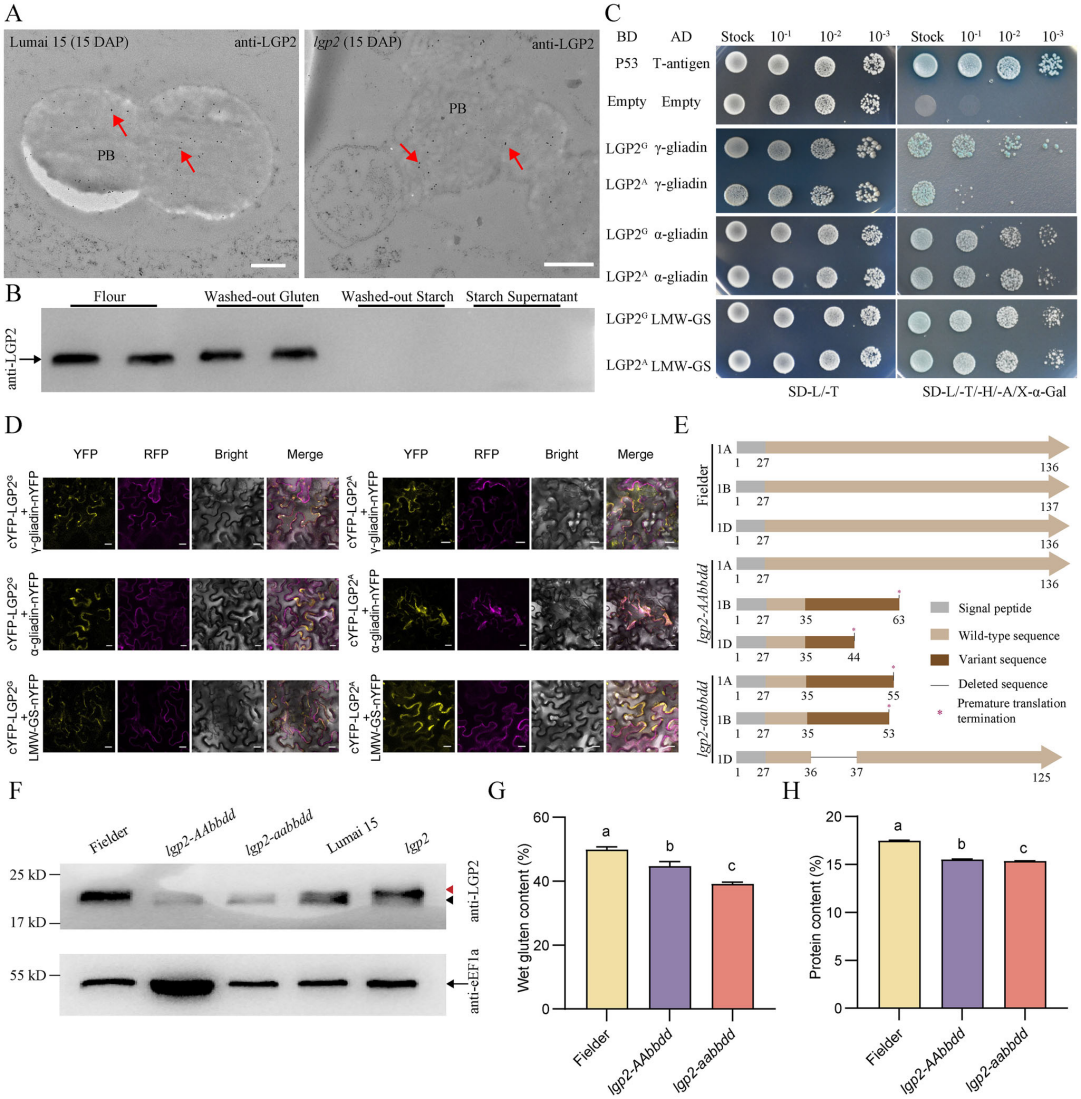
LGP2 is involved in gluten formation. (A) Representative TEM images showing immunogold labeling using anti‐LGP2 antibody in 15‐DAP Lumai 15 (top) and *lgp2* (bottom) starchy endosperm cells. PB, protein body. Red arrows indicate gold particles. Scale bars: 500 nm. (B) Immunoblot analysis of flour, washed‐out gluten, washed‐out starch, and supernatant liquid from washed starch, all derived from Lumai 15 seeds. (C) Yeast two‐hybrid assay showing interactions of LGP2^G^ and LGP2^A^ with seed storage proteins. LGP2^G^ and LGP2^A^ were expressed in the pGADT7 vector, and γ‐gliadin, α‐gliadin, and LMW‐GS were expressed in the pGBKT7 vector. Combinations of P53 and T‐antigen were used as positive control. (D) Bimolecular fluorescence complementation (BiFC) assays of the interactions between LGP2^G^/LGP2^A^ and seed storage proteins in *N. benthamiana* leaves. LGP2^G^ and LGP2^A^ were expressed in the pSAT1‐nYFP‐C1 vector, and γ‐gliadin, α‐gliadin, and LMW‐GS were expressed in the pSAT1‐cYFP‐C1‐B vector. The fused protein RFP‐HDEL was used as a positive control for ER localization. Scale bars: 20 µm. (E) Amino acid sequence alignment of LGP2 homeologs in Fielder and the knockdown lines *lgp2‐AAbbdd* and *lgp2‐aabbdd*. (F) Immunoblot analysis of the knockdown lines. Black arrowhead, wild‐type LGP2; red arrowhead, mutant LGP2; black arrow, eEF1a. (G,H) Wet gluten content (G) and protein content (H) of the *LGP2* knockdown lines. Data are means ± s.d. (*n* = 3 biologically independent samples). Group differences were assessed by one‐way ANOVA followed by Tukey's Honestly Significant Difference test. Different letters indicate significant differences at *P* < 0.05. Source data are provided as a Source Data file.

LGP2 accumulated strongly in grains and was located in protein bodies, as shown by immunoelectron microscopy using an anti‐LGP2 antibody (Figure 4A). We examined whether LGP2 is incorporated into the gluten network by interacting with glutenin and gliadins, as *lgp2* exhibited weakened gluten (Figure 1C). When we separated gluten and starch from Lumai 15 dough, LGP2 was exclusively detected in the gluten fraction, not in the starch or starch solution, as determined by immunoblotting using anti‐LGP2 antibody (Figure 4B). In yeast two‐hybrid and Bimolecular fluorescence complementation (BiFC) assays, both Lumai 15 LGP2 (LGP2^G^) and mutant LGP2 (LGP2^A^) interacted with seed storage proteins, including LMW‐GS, γ‐gliadin, and α‐gliadin (Figure 4C,D; Figure S8), suggesting that LGP2 might be associated with glutenins and gliadins. These results suggest that LGP2 is incorporated into the gluten network by interacting with glutenins and gliadins.

To investigate the roles of LGP2 in gluten formation and end‐use quality, we used CRISPR/Cas9‐mediated genome editing to target the first exon of the three homoeologous *LGP2* genes in wheat cultivar Fielder (Figure 4E; Figure S9). We obtained two homozygous genome‐edited lines with mutations in *LGP2* that led to premature termination codons or amino acid deletions. The line *lgp2‐aabbdd* contained knockout mutations in *LGP2‐1A* and *LGP2‐1B* as well as *LGP2‐1D*, leading to the production of LGP2 with an 11‐amino‐acid deletion (Figure 4E). The line *lgp2‐AAbbdd* contained knockout mutations in both *LGP2‐1B* and *LGP2‐1D*. We confirmed these results through immunoblotting using anti‐LGP2 antibodies (Figure 4F). Owing to the knockdown of *LGP2* in these two genome‐edited lines, the gluten and protein content were lower in the mutants than in Fielder (Figure 4G,H).

We heterologously expressed the *LGP2‐1D^G^* coding sequence driven by the endosperm‐specific glutenin gene promoter (*Pro^Glu^*) in rice (*Oryza sativa* ssp*. japonica*) variety Nipponbare. Five transgenic rice lines heterologously expressing *LGP2* (*Pro^Glu^:LGP2‐1D^G^#1/#2/#3/#4/#5*) showed greater protein content than non‐transgenic Nipponbare (Figure S10).

### 2.7 *lgp2* Flour Enhances Cookie Quality

**FIGURE 5:**
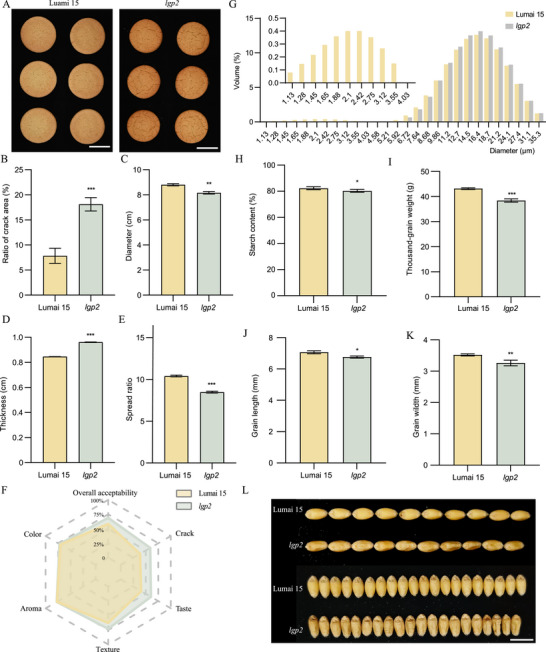
*lgp2* flour shows better cookie‐making performance than Lumai 15 flour. (A) Morphological differences in cookies made with flour derived from Lumai 15 or *lgp2* grains. Scale bars: 5 cm. (B–E) Ratio of crack area (B), diameter (C), thickness (D), and spread ratio (E) of cookies made with Lumai 15 or *lgp2* flour. Data are means ± s.d. (*n* = 3 or 6 biologically independent samples). (F) Sensory analysis performed on cookies made with Lumai 15 or *lgp2* flour. (G) Starch granule size distribution of Lumai 15 and *lgp2*. (H) Starch content of wheat flour of Lumai 15 and *lgp2*. (I‐K) Thousand‐grain weight (I), grain length (J), and (K) grain width of Lumai 15 and *lgp2*. (L) Grain morphology of Lumai 15 and *lgp2*. Scale bars: 1 cm. In (H–K), Data are means ± s.d. (*n* = 3 biologically independent samples). *p* values are from two‐sided Student's *t*‐tests (^*^p  < 0.05, ^**^p  < 0.01 and ^***^p  < 0.001). Source data are provided as a Source Data file.

Finally, we examined the performance of *lgp2* flour in cookie production, noting its weak gluten network (Figure 5A). For cookies, it is essential to achieve a significant spread with a large diameter and low thickness and to ensure a uniform surface‐cracking pattern [29]. Cookies made with *lgp2* flour exhibited a greater degree of cracking than cookies made with Lumai 15 flour (Figure 5B), suggesting that *lgp2* cookies had a more appealing surface. However, these cookies had a smaller diameter (Figure 5C), were thicker (Figure 5D), and displayed a lower spread ratio (Figure 5E), likely owing to the greater hardness of the kernels [6]. To evaluate consumer perceptions, we conducted sensory evaluations, including the appearance (crack), taste, texture, aroma, color, and overall acceptability of both Lumai 15 and *lgp2* cookies (Table S4). The *lgp2* cookies scored higher in terms of cracking, taste, and texture; specifically, cookies made with *lgp2* flour featured a deeper and more uniform cracking pattern on the surface, a crisper texture, consistent thickness, and a finer, more uniform internal structure (Figure 5F). These findings suggest that the *lgp2* mutation has the potential to enhance cookie quality, especially in terms of crack and taste, thereby catering to the preferences of specific markets and consumer groups. However, the improvement in cookie quality conferred by *LGP2* mutation comes with changed starch properties and a small penalty in yield. The *lgp2* mutant exhibited an increased proportion of A‐type starch granules, an almost complete absence of B‐type granules, and a reduced starch content (Figure 5G,H). Additionally, the *lgp2* mutant and genome‐edited lines show lower thousand‐grain weight and shorter grain width and length than Lumai 15 (Figure 5I–L; Figure S11A–C).

## 3 Discussion

### 3.1 Dual Genetic Strategies for Regulating Purothionin Production to Modulate Gluten Quantity and Quality in Wheat Flour

**FIGURE 6:**
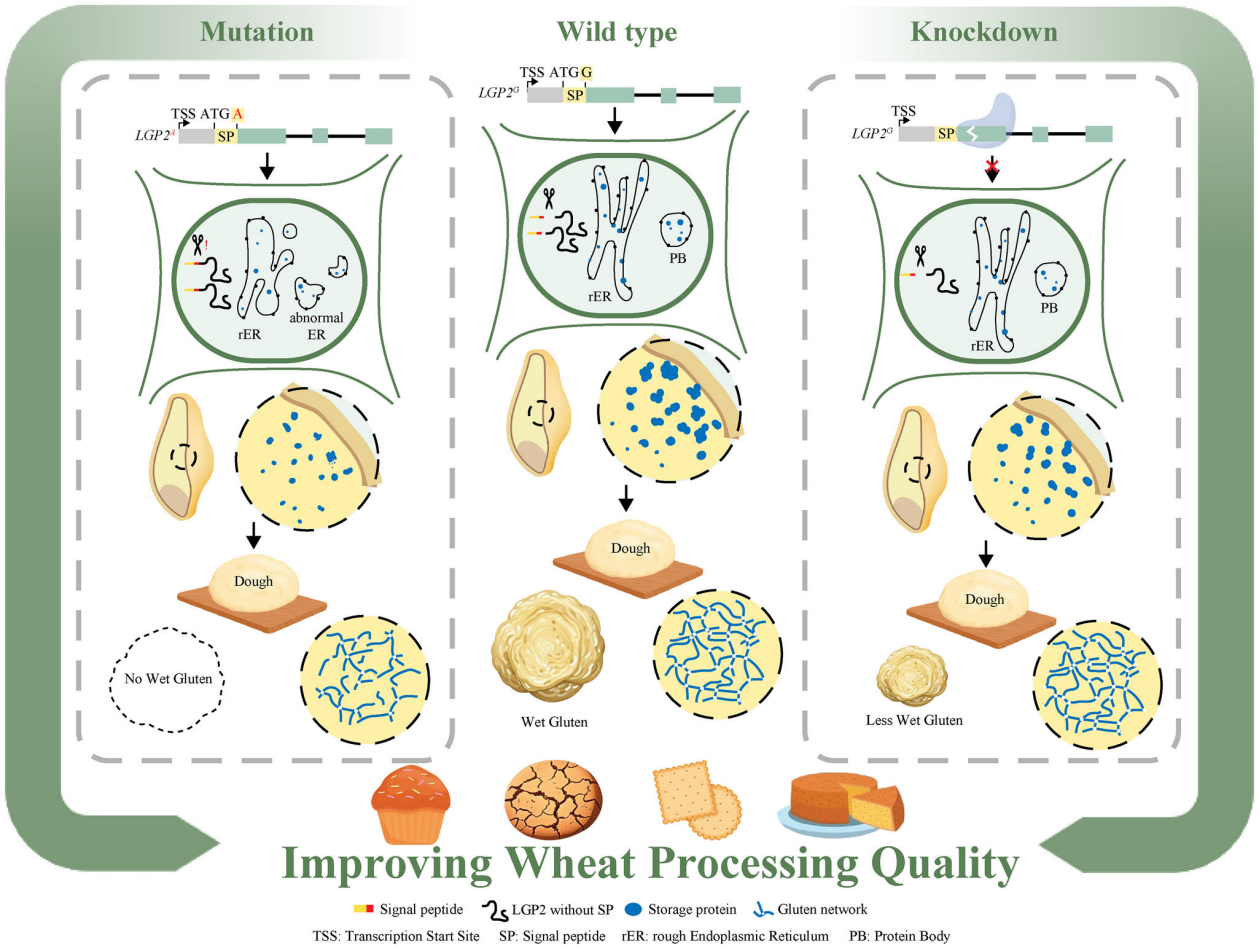
Dual genetic strategies for improving wheat processing quality by regulating purothionin accumulation to modulate gluten quantity and quality. The first strategy involves targeting SP cleavage sites via the mutation of *LGP2*, leading to ER stress and abnormal PB formation in wheat seeds, thereby reducing glutenin and gliadin accumulation and ultimately disrupting gluten network formation in dough. The second strategy involves modulating purothionin gene expression via knockdown, resulting in slightly lower protein content in seeds and weak gluten in dough. Both techniques improve wheat processing quality for cookie making. TSS, transcription Start Site; SP, signal peptide; rER, rough endoplasmic reticulum; PB, protein body.

In this study, we identified the wheat *lgp2* mutant, which has lower gluten content (Figure 1A), weakened gluten network strength (Figure 1B,C), and improved cookie‐making performance compared with Lumai 15 (Figure 5A,B and F). This mutant harbor a missense mutation in the region encoding the SP cleavage site of alpha‐2‐purothionin protein. The presence of the unprocessed protein leads to ER stress (Figure 3A–C; Figure S5) and the formation of abnormal protein bodies (Figure 3D,E). We discovered that alpha‐2‐purothionin plays a role in gluten formation by interacting with seed storage proteins. Knocking down *LGP2* resulted in lower gluten content (Figure 4F) as well as lower protein content (Figure 4G). Based on these findings, we propose two strategies for enhancing the processing quality of speciality wheat for low‐gluten food production (Figure 6). The first strategy involves targeting SP cleavage sites (e.g., through mutation) to indirectly reduce gluten content, thereby disrupting gluten network formation. The second strategy focuses on modulating purothionin gene expression (e.g., via knockdown or knockout) to fine‐tune both the quantity and quality of gluten.

### 3.2 The Role of Purothionin in Wheat End‐Use Quality

Purothionin, originally isolated from wheat flour, is a member of the thionin protein family. This family has been identified in both cereal and non‐cereal crops [30, 31]. Various studies have focused on the roles of thionins as plant antimicrobial peptides because of their antimicrobial properties. The toxicity of purothionin has been observed in bacteria, fungi, and insects [32, 33, 34]. For example, applying purothionin to wheat enhances its defenses against leaf rust, demonstrating the potential of purothionin for improving disease resistance through molecular breeding [35]. Additionally, purothionin exhibits thioredoxin‐like activity, playing a role in seed germination [36, 37]. In the current study, we detected purothionin in gluten networks and determined that it functions in the gluten network by interacting with gliadins and glutenins. Purothionin contains eight half‐cystine residues that enable the formation of disulfide bonds [31], which are crucial for establishing protein linkages during gluten formation. The finding that *lgp2* gene‐edited knockdown lines exhibit lower wet gluten and protein content than the wild type supports the notion that purothionin plays important roles in gluten formation beyond plant defense.

### 3.3 Incorporating Storage Proteins with Unprocessed Signal Peptides is an Effective Strategy for Developing Wheat Varieties With Low Gluten Content

Impaired SP cleavage of storage proteins leads to ER stress, the unfolded protein response, and defective PB formation, as demonstrated in maize (*Zea mays*), sorghum (*Sorghum bicolor*), and wheat [16, 38, 39, 40, 41, 42]. The maize mutants *fl2* (*floury4*), DE^*^‐B30 (defective endosperm^*^‐B30), and *fl4* (*floury4*), containing mutated α‐zein with unprocessed SPs, exhibit a disrupted PB pathway, leading to opaque endosperm, lower kernel weight, and greater lysine content [39, 40, 43]. A high‐lysine sorghum mutant characterized by floury kernels displays deformed PBs caused by an amino acid substitution at the SP cleavage site of α‐kafirin [41]. In the wheat mutant *lgp1*, defective SP cleavage within a gliadin impairs PB formation and simultaneously leads to lower levels of both glutenin and gliadin via a shared mechanism, leading to dough that fails to form gluten [16]. Here, we demonstrated that unprocessed purothionin induces ER stress and abnormal PB formation, leading to diminished glutenin and gliadin accumulation and weak gluten, which is similar to the effect of unprocessed gliadin. Therefore, modulating the cleavage of the SPs of storage proteins in wheat offers a promising strategy for producing weak‐gluten wheat.

### 3.4 Potential Utilization of the Purothionin Gene During Wheat Breeding

Breeding for functional and end–use‐specific wheat varieties has garnered increased attention [44]. Patients with certain medical conditions must manage their gluten and protein intake; therefore, low‐gluten wheat varieties could be beneficial by offering reduced antigen levels while allowing for a relatively normal diet [45, 46, 47]. The *lgp2* allele may serve as a novel genetic resource for breeders aiming to breed low‐protein wheat varieties.

We suggest dual genetic strategies that use purothionin gene alterations to enhance cookie quality, but the significant yield loss associated with this approach requires further investigation to optimize its application. For instance, the maize *fl2* mutant exhibits lower kernel weight than wild type, but its yield can be restored to 80–90% of that of wildtype by backcrossing the *fl2* allele into other genetic backgrounds [48, 49]. Similarly, hybridization with different high‐yield varieties was conducted to address the 25% reduction in kernel weight observed in a sorghum mutant, resulting in a single‐cross hybrid that produced 35% more lysine without compromising yield [50]. In the current study, *lgp2* showed 11% lower yield than Lumai 15, which was only 8% after backcrossing to other backgrounds (data not shown). Thus, evaluating the *lgp2* allele in various genetic backgrounds is crucial for minimizing yield penalties. Compared with *LGP1*, a previously reported gene whose mutation leads to weak gluten with almost 50% yield penalty, *LGP2* is a better candidate for quality improvement and breeding applications. Notably, additional autophagy or programmed cell death has not been observed in the *lgp2* mutant, but is prevalent in *lgp1*. This indicates that ER stress is less severe in *lgp2*, perhaps explaining the lower yield loss associated with this mutant [16]. In the future, we plan to develop genetic materials with varying levels of *LGP2* expression to investigate its effect on the gluten network or to use prime editing to precisely manipulate the number of disulfide bonds in LGP2. This will allow us to explore the potential of targeting purothionin to fine‐tune gluten strength in wheat flour.

## 4 Experimental Section

### 4.1 Plant Materials and Growth Conditions

The EMS‐treated wheat (*Triticum aestivum* L.) population used for mutant screening was the soft wheat cultivar Lumai 15, with a high‐molecular‐weight glutenin subunit (HMW‐GS) profile of 1Ax1, 1Bx7 +1By8, 1Dx2 + 1Dy12. The M_5_ generation was grown in HanDan, HeBei, China (36°34′N, 114°28′E) in 2019, and gluten levels were measured in mature seeds.

For map‐based cloning, F_2_ populations were generated from crosses between *lgp2* and common wheat cultivar Nongda 3331 (ND3331), with the following HMW‐GS profile: 1Ax null, 1Bx17 +1By18, 1Dx2 + 1Dy12. To evaluate the effects of *lgp2* in different genetic backgrounds, three BC_2_F_2_ populations were generated using three reciprocal parents: Lumai 15, Jing 411, and Zhengmai 50 (Jing 411 and Zhengmai 50 were also soft wheat cultivars). The plants were grown in HanDan, HeBei, China, from 2020 to 2024.

Transgenic wheat lines and the corresponding non‐transgenic cultivar Fielder were grown in Shangzhuang Experimental Station at China Agricultural University in Beijing, China (39°54′N, 116°23′E) for phenotypic analysis. Transgenic rice (*Oryza sativa*) lines and the corresponding non‐transgenic cultivar Nipponbare were grown in paddy fields in Hainan, China (18°48′N, 110°02′E).

### 4.2 Phenotypic Analysis

Glutenins and gliadins were extracted from the samples and separated as described [51]. The microstructure of dough samples was observed using confocal laser scanning microscopy according to [52]. Reversed‐phase high‐performance liquid chromatography (RP‐HPLC) and quality analysis were conducted as described previously [53]. The kernel hardness index was measured using the Perten Single Kernel Characterization System (SKCS) 4100 (Perten Instruments North America, Inc., Reno, NV) using randomly selected seeds. Images of mature wheat endosperm were taken under a Hitachi S‐3400N scanning electron microscope (Tokyo, Japan). Grain length, grain width, and thousand‐grain weight were assessed in mature seeds using a Wanshen SC‐G Seed Detector (Wanshen Detection Technology, Hangzhou, China). Wet gluten content was determined by AACC Method 38‐12.02 [54]. At least three biological replicates were conducted.

### 4.3 Map‐Based Cloning

The F_2_ population of *lgp2* × ND3331 was used to map the *LGP2* gene controlling gluten protein levels. For genome resequencing, genomic DNA samples from *lgp2* were sequenced with 6× coverage using the NovaSeq 6000 platform (Illumina). Reads for ND3331 were downloaded from NCBI (accession SRS5949726). High‐quality reads were aligned to the wheat reference genome (IWGSC RefSeq v.1.1). Insertion–deletion markers (designated with the prefix “ID‐”) and single‐nucleotide polymorphism (SNP) markers (designated with the prefix “SNP‐”) were developed based on the genomic resequencing data (Table S5). These markers were used to analyze 51 homozygous F_2_ plants showing the *lgp2* mutant phenotype and a larger F_2_ population of 1963 plants for mapping. The primers are listed in Table S5.

### 4.4 Signal Peptide Identification

The amino‐acid sequences of LGP2 from Lumai15 and the *lgp2* mutant (derived from Sanger sequencing) were submitted to the S_IGNAL_P 6.0 server. Predictions were run with the full (slow) model and the eukaryotic organism setting.

### 4.5 RNA‐Seq and Data Analysis

Total RNA was extracted from developing wheat seeds at 15 and 20 days after pollination (DAP) from *lgp2* and Lumai 15 using a TransZol Plant Kit (TransGen Biotech). RNA‐seq library construction was performed using a TruSeq RNA Sample Prep Kit v2 (Illumina), and transcriptome sequencing was conducted on the NovaSeq 6000 platform. RNA‐seq reads were aligned to the “Chinese Spring” reference genome (IWGSC RefSeq v1.1). Fragments per kilobase of transcripts per million mapped reads (FPKM) of each gene and differentially expressed genes (DEGs) were analyzed using DESeq2 v.1.24.0. Genes with log_2_(fold change) ≥ 0.8 and false discovery rate (FDR) < 0.05 were identified as DEGs. Three biological replicates were performed. The raw RNA‐seq reads have been deposited in the NCBI Sequence Read Archive (SRA) under BioProject number PRJNA1281312.

### 4.6 Expression Vector Construction and Transformation

For transient expression and subcellular localization, the full‐length coding sequences (CDSs) of *LGP2‐1D* (from both Lumai 15 and *lgp2* plants) and *PINa* and *PINb* (both from Lumai 15), all without termination codons, were cloned into the pCAMBIAsuper1300‐GFP vector [55].

To construct the *LGP2* overexpression vector, the full‐length CDSs of *LGP2‐1D^A^* amplified from developing *lgp2* seeds and *LGP2‐1D^G^* amplified from developing Lumai 15 seeds were cloned into a modified pMWB110 vector under the control of the *lgp1* promoter from the wheat gliadin gene and the *Glu‐1Dy10* promoter from the wheat glutenin gene [16, 56]. To construct the prime‐editing vector, a prime‐editing guide RNA (pegRNA) was designed to edit the *LGP2* sequence. The reverse‐transcription template was 16 bp long and had a G‐to‐A substitution that converts Gly27 to Asp. The spacer sequence for spCas9 and the primer binding site were 20 bp and 10 bp long, respectively, targeting both *LGP2‐1A* and *LGP2‐1D*. The pegRNA was cloned into the pBUE414‐ePPEmax‐V223A vector as described previously [16, 57]. To construct the CRISPR/Cas9 gene‐editing vector, a single guide RNA (sgRNA) was designed within the CDSs of *LGP2* homoeologs using the website http://www.e‐crispr.org/E‐CRISP/designcrispr.html and inserted into the expression cassette of the pBUE411 vector [16, 58].

All constructs were introduced into *Agrobacterium tumefaciens* strain EHA105 and transformed into wheat cultivar Fielder or rice cultivar Nipponbare. All primers are listed in Table S6.

### 4.7 Antibodies

Anti‐GFP (HT801‐01; TransGen Biotech), anti‐BiP (AS09 481; Agrisera), anti‐eEF1α (AS10 934; Agrisera), and anti‐rabbit IgG (BE0101‐100; Easybio) antibodies were used at a 1:5000 dilution for immunoblot analysis. Anti‐LGP2, anti‐PINa, and anti‐PINb antibodies were synthesized by ABclonal and used at a 1:5000 dilution for immunoblot analysis. Anti‐LGP2 and anti‐rabbit IgG antibodies were used at a 1:100 dilution for immunogold labeling.

### 4.8 Immunoblot Analysis

Endosperm was collected from Lumai 15 and *lgp2* grains at 25 DAP and from mature grains of Lumai 15, *lgp2*, Fielder and the transgenic lines. Flour, dough, starch, and starch solution from Lumai 15 were prepared as previously described [59]. Protein extraction and immunoblot analysis were performed as previously described [60].

### 4.9 Subcellular Localization

LGP2‐1D^G^‐GFP (Lumai 15), LGP2‐1D^A^‐GFP (*lgp2*), PINa‐GFP, and PINb‐GFP were co‐expressed with the ER marker RFP‐HDEL in wheat leaf protoplasts of Fielder as previously described [61]. GFP and RFP signals were imaged 16 h after transformation. GFP and RFP signals were imaged under a confocal microscope (LSM880; Carl Zeiss, Heidenheim, Germany; laser at 488 nm for GFP and 543 nm for RFP).

### 4.10 Transmission Electron Microscopy (TEM)

For conventional chemical fixation, transverse sections of 12‐DAP fresh seeds were fixed in 0.5% (v/v) glutaraldehyde and 3% (w/v) paraformaldehyde, followed by 2% OsO_4_, then dehydrated, embedded in LR White Resin (London Resin, Berkshire, UK), and cut with a tissue slicer (RM2265; Leica). For high‐pressure freezing (HPF), 12‐DAP fresh seeds were frozen in an HPF apparatus (EM PACT2; Leica); infiltration with Lowicryl HM20 (Electron Microscopy Sciences, Hatfield, PA, USA), embedding, and UV polymerization were performed stepwise at −35°C. For immunogold localization, the sections were blocked with 1% BSA for 30 min, incubated with primary and secondary antibodies for 2 h each at room temperature, double‐stained with 2% (w/v) uranyl acetate and 2.5% (w/v) lead citrate, and examined under a Hitachi H‐7600 transmission electron microscope. Three individual replicate seeds were analyzed.

### 4.11 Y2H Assay

The CDSs of *LGP2‐1D^G^* (Lumai 15) and *LGP2‐1D^A^* (*lgp2*) were cloned into the pGBKT7 vector (Takara, Japan) as bait, and the CDSs of other seed storage proteins were fused to pGADT7 as prey (Takara, Japan). Different combinations of plasmids were transformed into the yeast (*Saccharomyces cerevisiae*) strain AH109. All primers are listed in Table S6.

### 4.12 BiFC Assay

The BiFC assay was performed by using n‐YFP and c‐YFP vectors harboring the fragments encoding the N‐ and C‐terminal halves of yellow fluorescent protein (YFP), respectively. The CDSs of LGP^G^ and LGP^A^ were cloned into the pSAT1‐nYFP‐C1 vector, and γ‐gliadin, α‐gliadin, and LMW‐GS were cloned into the pSAT1‐cYFP‐C1‐B vector. The combinations of plasmids were transformed into the *A. tumefaciens* strain GV3101 and co‐expressed in *N. benthamiana* leaves. The fused protein RFP‐HDEL was used as a positive control for ER localization. The YFP signal was imaged after 48 h of infiltration using a confocal microscope (LSM900; Carl Zeiss). The primer sequences are listed in Table S6.

### 4.13 Cookie Preparation and Evaluation

Cookies were prepared according to AACC Method 10–52 with some modifications. The ingredients used were flour (225 g), sugar (135 g), shortening (67.5 g), nonfat dry milk (6.75 g), and sodium bicarbonate (3.25 g). The dough was sheeted to a 7‐mm thickness on a dough sheeter and cut into circles using a 60‐mm‐diameter cutter. Baking was performed in a baking oven at 205°C for 15 min. The diameter and thickness of six cookies were measured, and the mean was calculated. The spread ratio was calculated by dividing the diameter (mm) by the thickness (mm).

To identify and quantify crack areas, cookie images were processed using ImageJ software. The original image was processed using the Canny algorithm for edge detection, followed by the Threshold, Fill Holes, and Convex Hull tools. Crack areas and surface areas were then calculated using the Analyze Particles tool. The crack ratio was calculated by dividing the crack area by the surface area.

For sensory evaluation, 18 experienced consumers (9 women and 9 men) aged 22 to 65 years old participated in the study. All evaluations were conducted at room temperature on the same day. Cookies were prepared 1 day before sensory evaluation and stored at room temperature. Samples were cut into standard sizes (5 g) and presented to each assessor in random order. Sensory properties (crack, taste, texture, aroma, color, and overall acceptability) were evaluated. Scoring criteria are listed in Table S4.

## Author Contributions

Y.Y. and Q.S. conceived the project. Y.L., S.C., and X.Z. performed the majority of experiments. T.K. made the cookie and evaluated its performance. F.N. and Y.B. performed the EMS population generation. M.L. and Z.Z. performed bioinformatics analysis; Y.Z. and J.L. provided technological assistance in gene editing. M.X., J.D., Z.H., R.Z., and Z.N. provided contributions to data analysis and writing. All authors agreed to the final version of the manuscript.

## Conflicts of Interest

The authors declare no conflicts of interest.

## Supporting information




**Supporting File 1**: advs73696‐sup‐0001‐SuppMat.pdf.

**Supporting File 2**: advs73696‐sup‐0002‐Data.zip.

**Supporting File 3**: advs73696‐sup‐0003‐Supplemental Tables.xlsx.

## Data Availability

The data that support the findings of this study are available in the supplementary material of this article.
