# Supplementary material for: Modulating Purothionin Accumulation and Signal Peptide Cleavage Fine‐Tunes Wheat Flour Gluten Properties to Improve Cookie‐Making Quality
Source: Adv Sci (Weinh). 2026 Jan 7;13(16):e12581. doi: 10.1002/advs.202512581 (PMC13042976; doi:10.1002/advs.202512581)
Supplement: Supplementary file 1 — Supporting File 1: advs73696‐sup‐0001‐SuppMat.pdf. [file ADVS-13-e12581-s001.pdf]

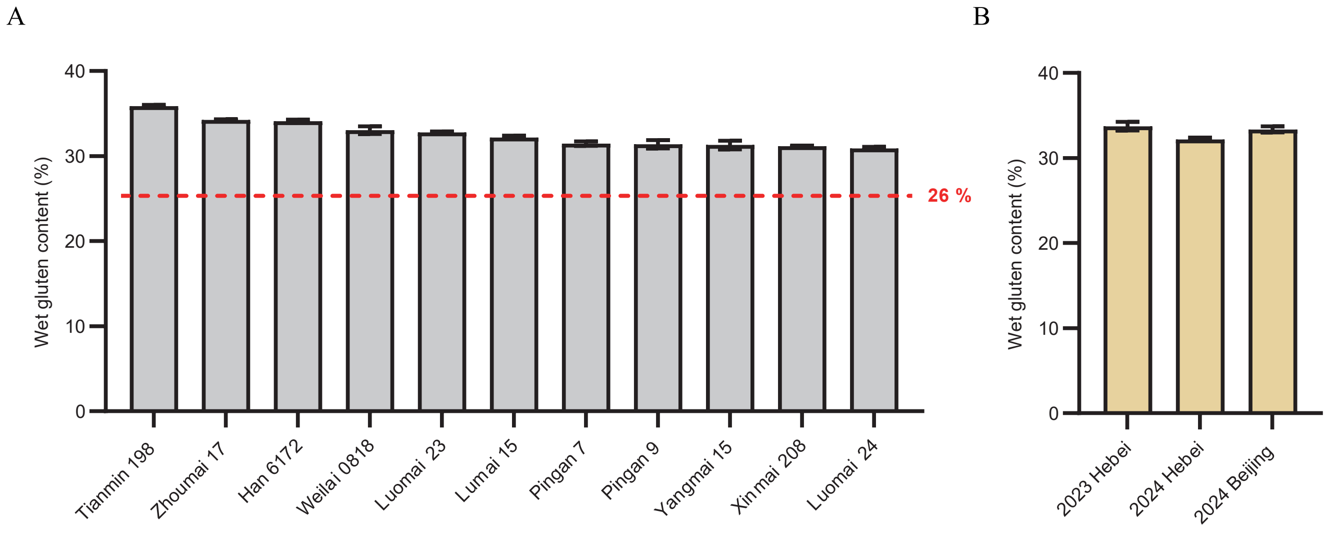

Figure S1. Comparison of wet gluten content among soft wheat varieties and environments.
(A) Comparison of wet gluten content among soft wheat varieties. The red dashed line represents the maximum wet gluten content required for cookie flour in China. **(B)** Wet gluten content of Lumai 15 grown in different environments. Data are means ± s.d. (*n* = 3 biologically independent samples).

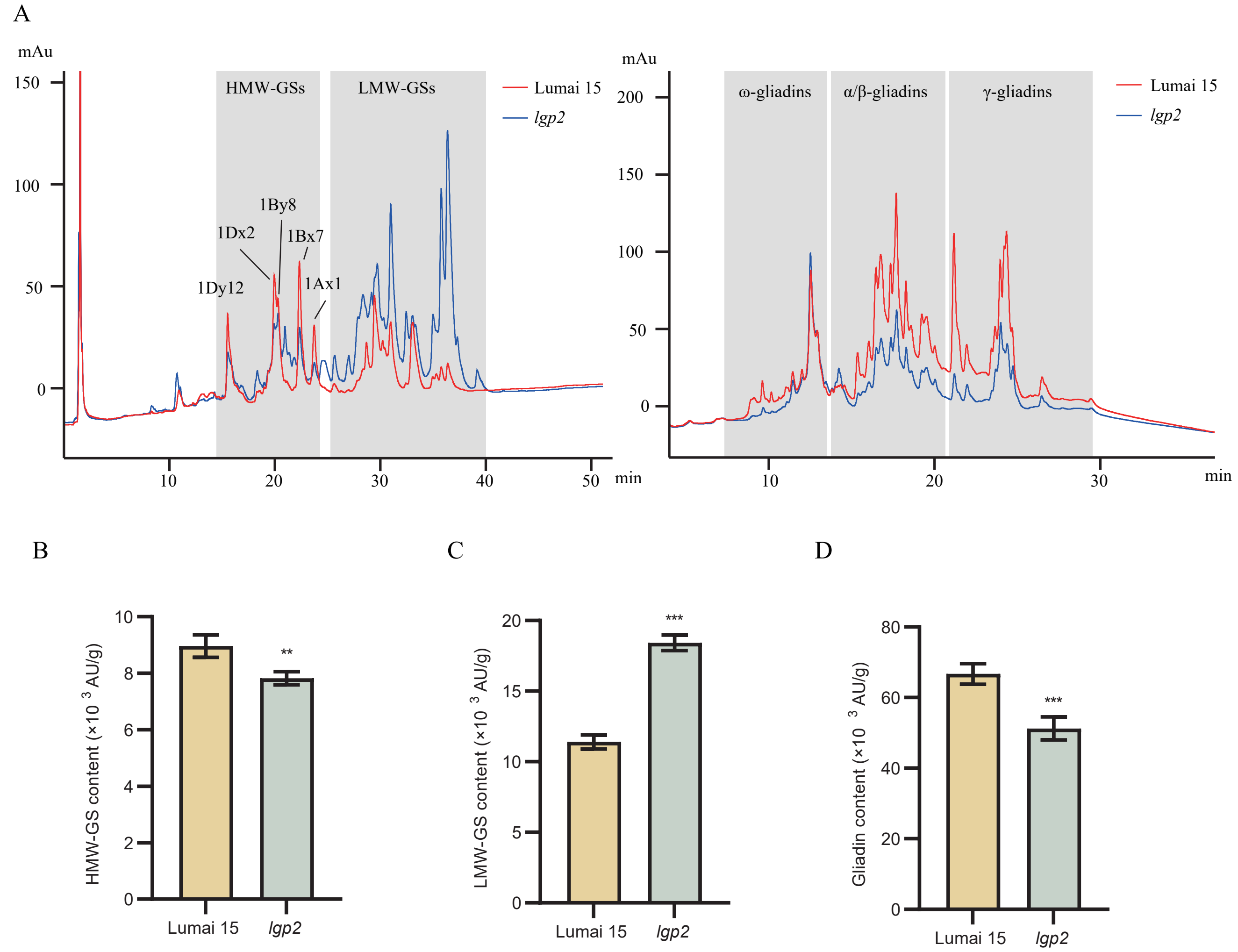

Figure S2. RP-HPLC chromatograms of Lumai 15 and *lgp2* extracts.
(A) Representative RP-HPLC chromatograms of glutenin and gliadin extracts from Lumai 15 (WT) and *lgp2*. The left panel shows the accumulation of HMW-GSs (high-molecular-weight glutenin subunits) and LMW-GSs (low-molecular-weight glutenin subunits). The right panel shows the accumulation of all types of gliadins. **(B–D)** Peak areas of HMW-GS **(B)**, LMW-GS **(C)**, and gliadin **(D)** content in RP-HPLC chromatograms of Lumai 15 and *lgp2* extracts. Data are means ± s.d. (n = 4 biologically independent samples). P values are from two-sided Student's t-tests (**P < 0.01 and ***P < 0.001). Source data are provided as a Source Data file.

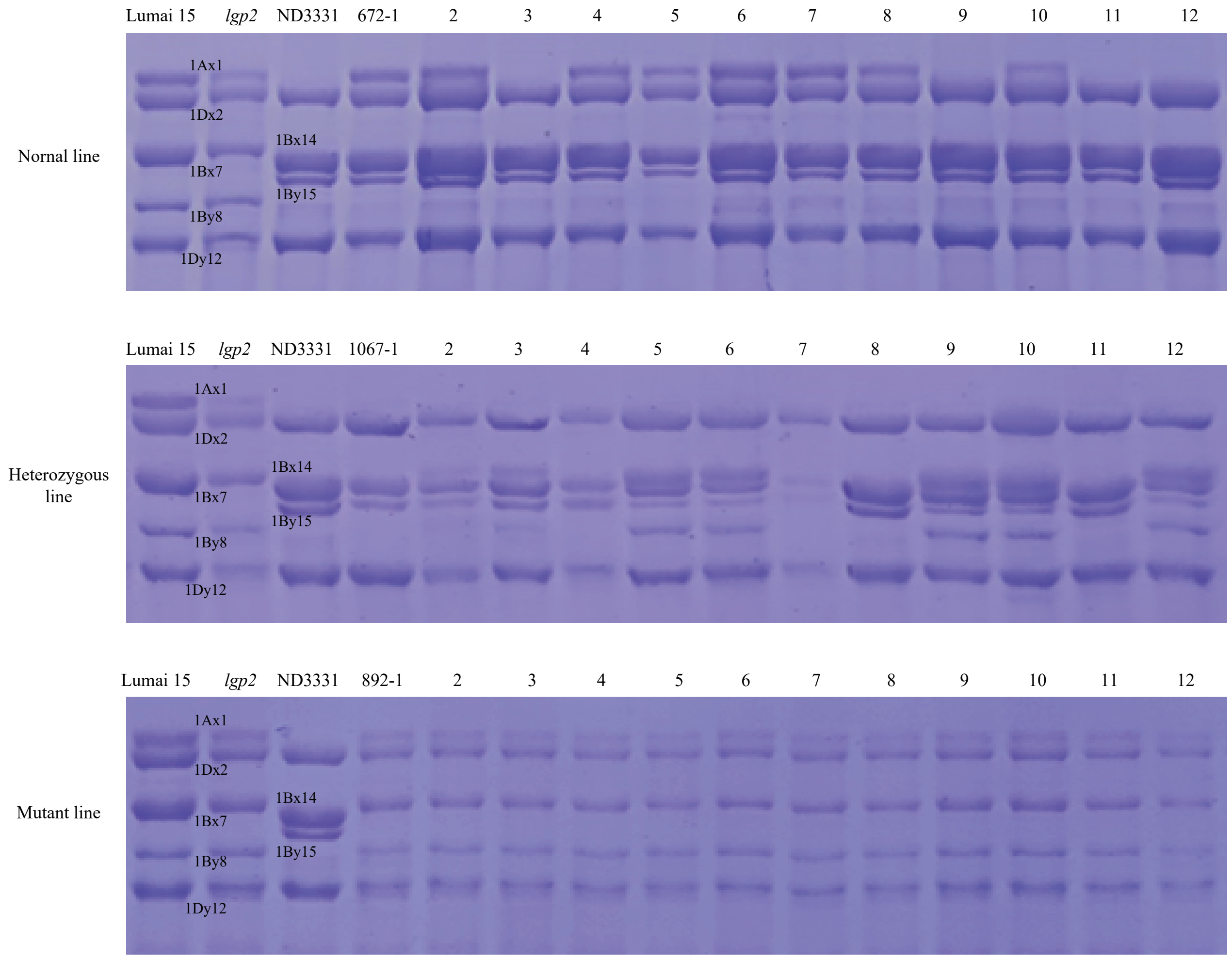

Figure S3. Phenotypic analysis of F_2 progenies from ND3331 × *lgp2* seeds.
HMW-GS levels of representative F_2 plants derived by self-pollination of ND3331 × *lgp2* detected by SDS-PAGE followed by Coomassie Blue staining. The first three lanes represent the parental lines: Lumai 15 and *lgp2* have HMW-GS compositions of 1Ax1, 1Bx7+1By8, and 1Dx2+1Dy12, while ND3331 displays 1Ax null, 1Bx14+1By15, and 1Dx2+1Dy12. Twelve progeny seeds generated from individual F_2 plants were examined; 672, 1067, and 892 are shown as examples. "Normal line" (such as 672) indicates a homozygous line in which all 12 seeds showed the same (high) HMW-GS level as the parent ND3331. "Mutant line" (such as 1067) indicates a homozygous line in which all 12 seeds showed the same (low) HMW-GS level as *lgp2*. In heterozygous lines (such as 892), the 12 progeny seeds showed segregation for HMW-GS content.

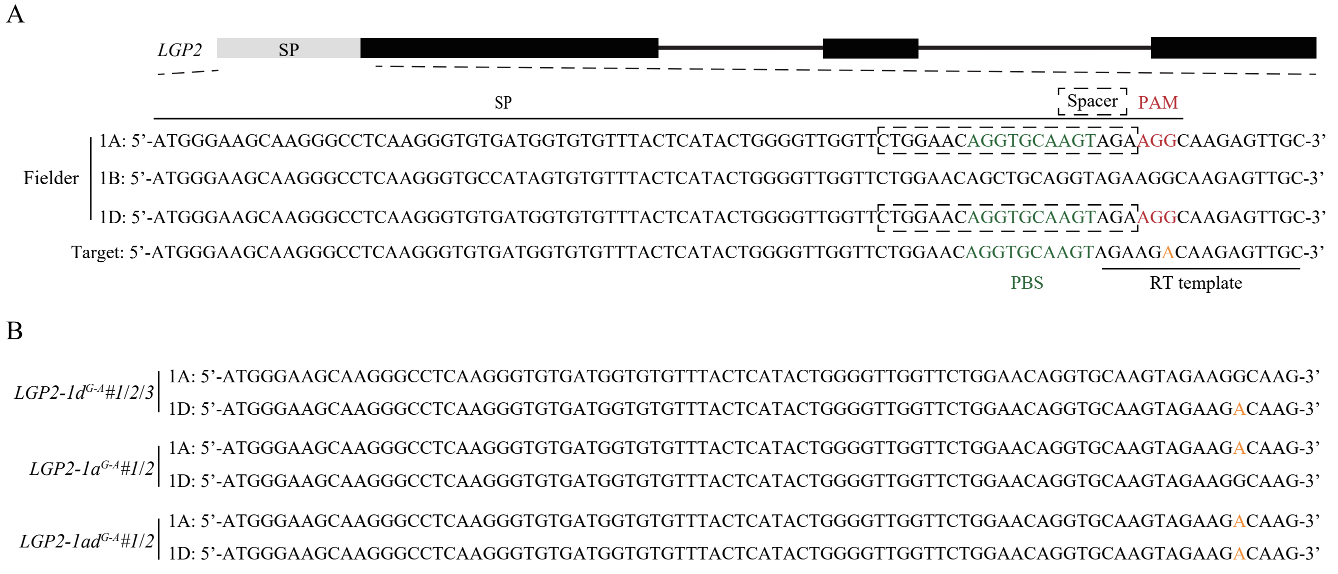

Figure S4. Gene structure of *LGP2*, with targets for CRISPR/Cas9-specific binding to the signal peptide coding region of this gene, and sequence alignment of *LGP2* in Fielder and the genome-edited lines.

(A) Gene structure of *LGP2* with a target for CRISPR/Cas9 and a primer binding site (PBS) for reverse transcriptase-specific binding to the C terminus of the signal peptide (SP). RT template, reverse-transcription template. **(B)** Alignment of signal peptides of *LGP2-1d*$^{G\text{-}A}$*#1/2/3*, *LGP2-1a*$^{G\text{-}A}$*#1/2*, and *LGP2-1ad*$^{G\text{-}A}$*#1/2*. The protospacer-adjacent motif (PAM) sequence is indicated in red. The spacer sequence for spCas9 is indicated in a dashed box. The PBS of the prime-editing guide RNA is indicated in green. The G-to-A mutation is indicated in yellow.In heterozygous lines (such as 892), the 12 progeny seeds showed segregation for HMW-GS content.

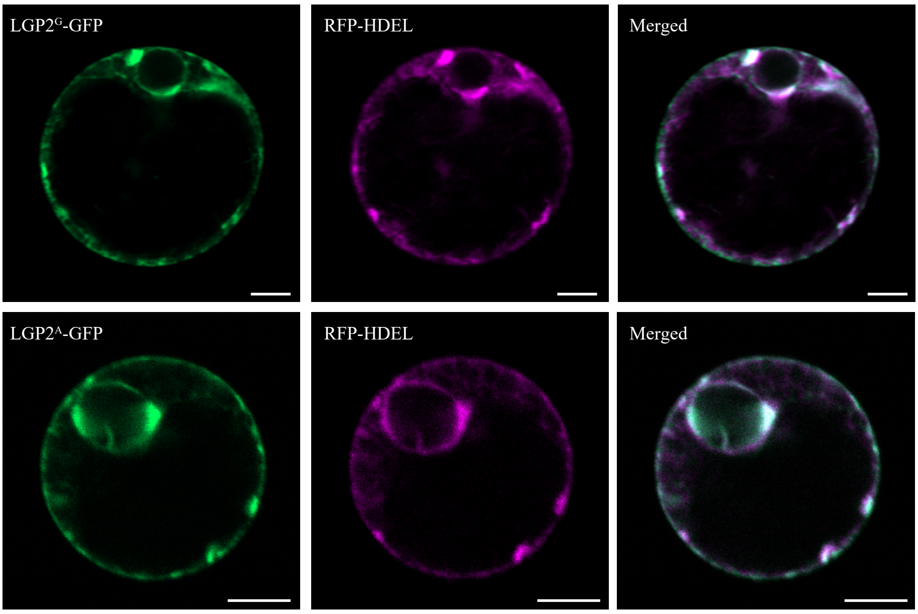

Figure S5. Subcellular localization of LGP2^{G} and LGP2^{A}.
Representative images of the subcellular localization of LGP2^{G}-GFP and LGP2A-GFP fusion proteins, which co-localized with the ER marker RFP-HDEL in wheat protoplasts of Fielder. Scale bars: 10 μm.

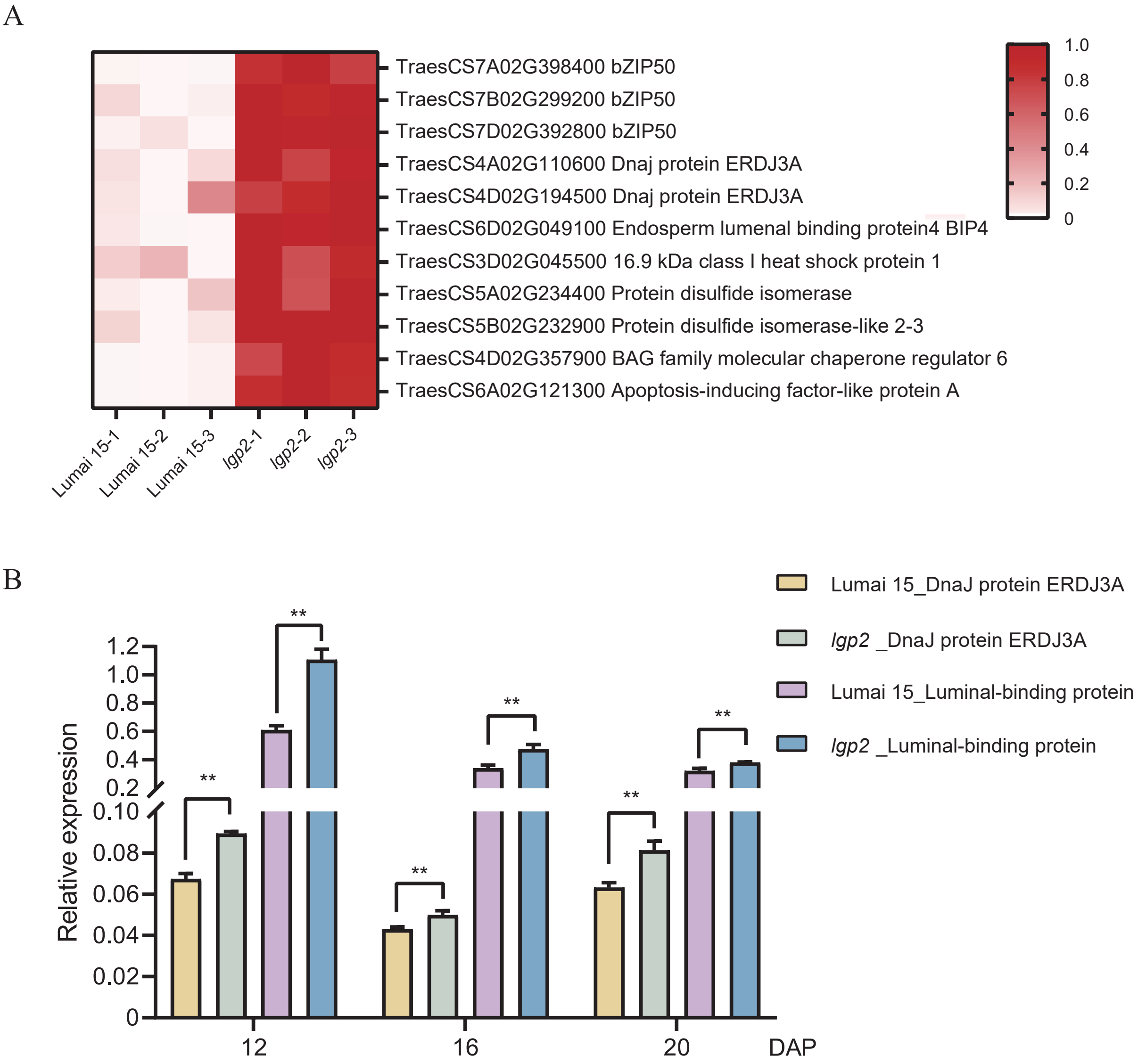

Figure S6. Expression of ER stress–associated genes in Lumai 15 and *lgp2*.
(A) Heatmap illustrating the FPKM-based expression patterns of ER stress–associated genes in 20-DAP endosperm of Lumai 15 and *lgp2*. For each gene, the average FPKM value of three biological replicates per sample was normalized by GraphPad Prism and reported in the heatmap. **(B)** Ralative expression of TraesCS4A02G110600 (DnaJ protein ERDJ3A) and TraesCS6D02G049100 (Luminal-binding protein 4) in devlopment seeds from Lumai 15 and *lgp2*. Datas were normalized to ACTIN. Data are the mean ± SD of $n = 3$ replicates. Statistically significant differences are indicated by **, $P < 0.01$, as determined by Student's t test.

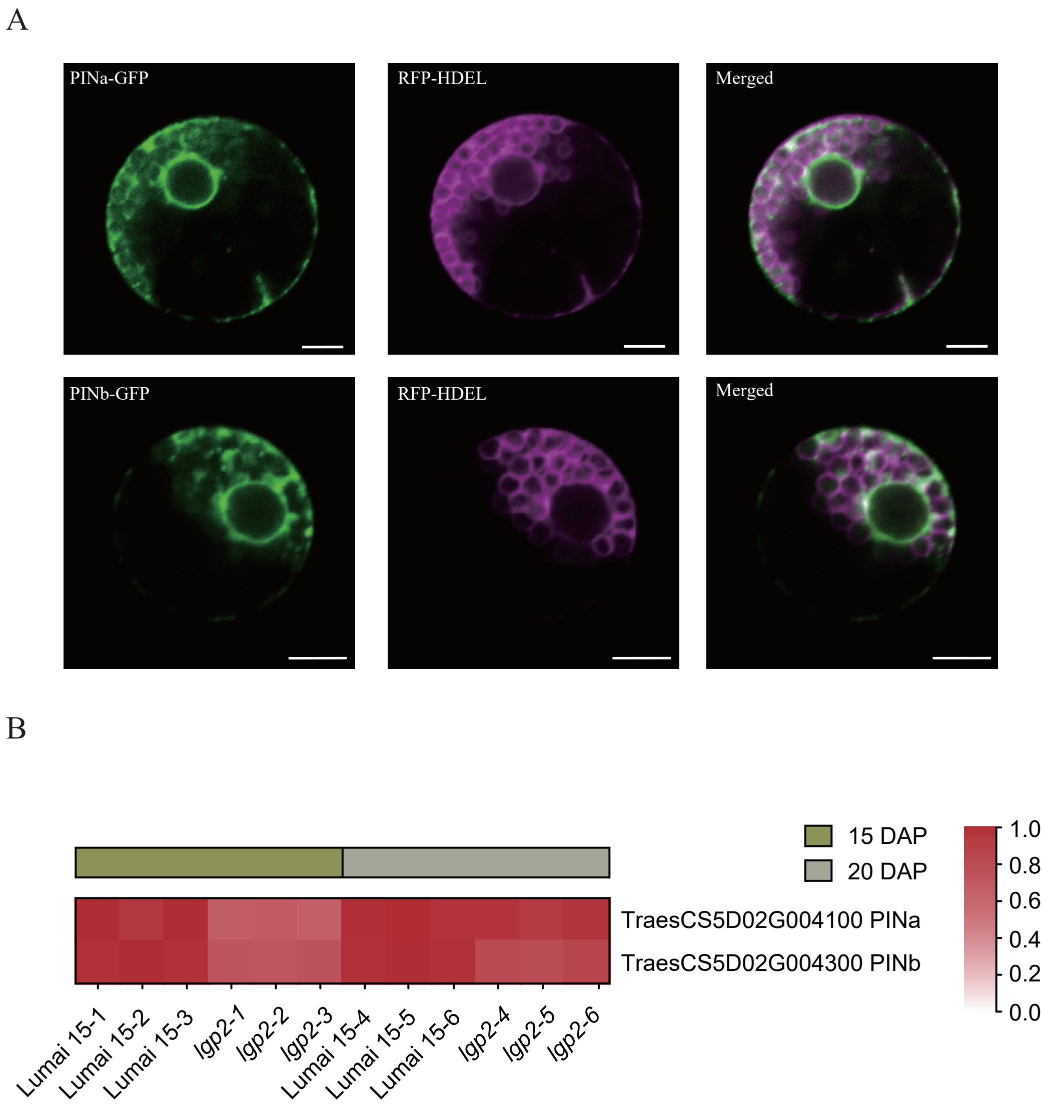

Figure S7. Subcellular localization of PINs and expession level of *PIN* genes.
(A) Representative images of the subcellular localization of PINa-GFP and PINb-GFP fusion proteins, which co-localized with the ER marker RFP-HDEL in wheat protoplasts of Fielder. Scale bars: 10 μm. **(B)** Heatmap illustrating the FPKM-based expression patterns of *PIN* genes in 15- and 20-DAP endosperm of Lumai 15 and *lgp2*. For each gene, the average FPKM value of three biological replicates per sample was normalized by GraphPad Prism and reported in the heatmap.

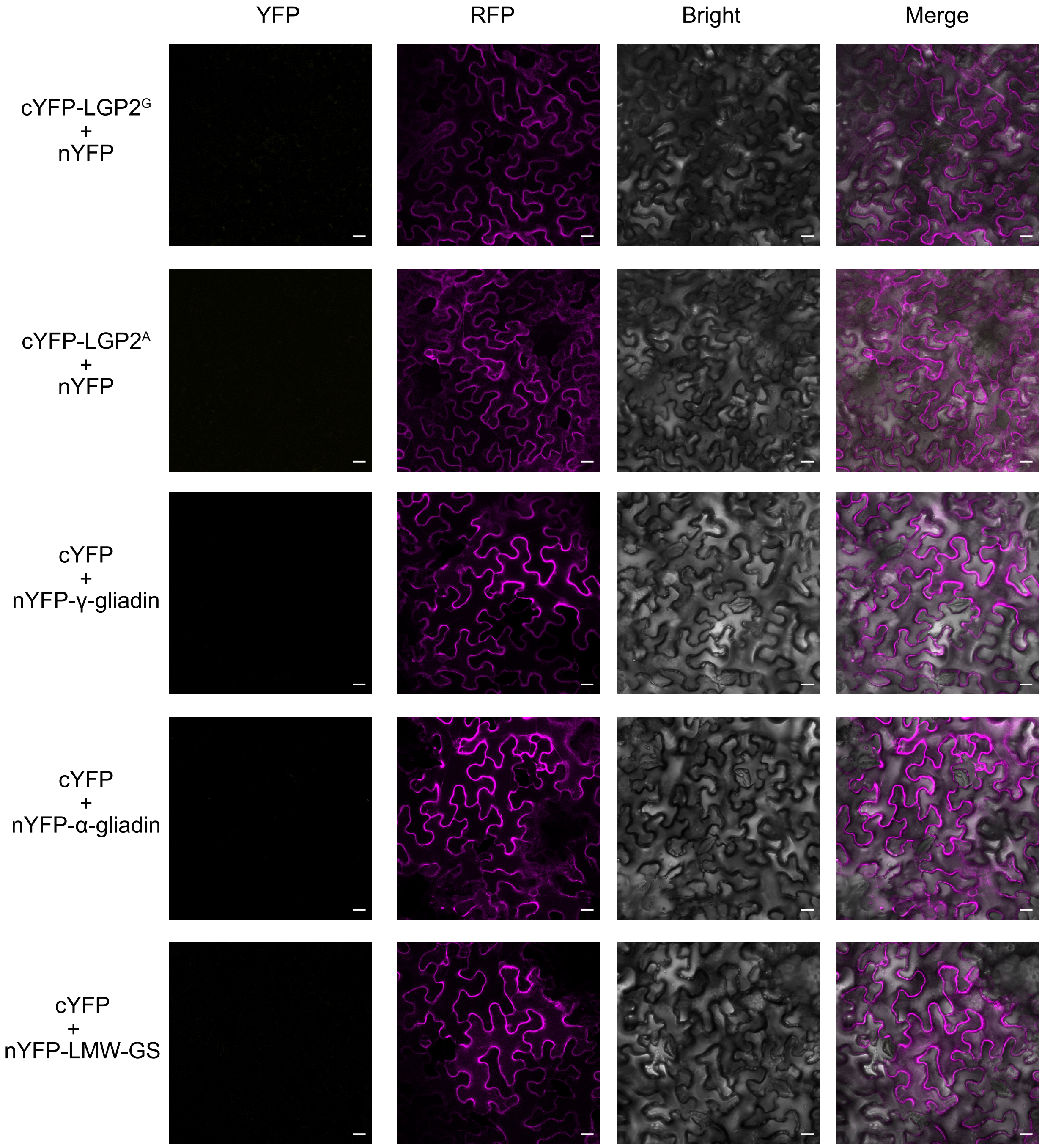

Figure S8. Bimolecular fluorescence complementation (BiFC) assays of the interactions between LGP2 and seed storage proteins in *N. benthamiana* leaves (negative control).
nYFPand cYFP was used as negative control. The fused protein RFP-HDEL was used as a positive control for ER localization. Scale bars: 20 μm.

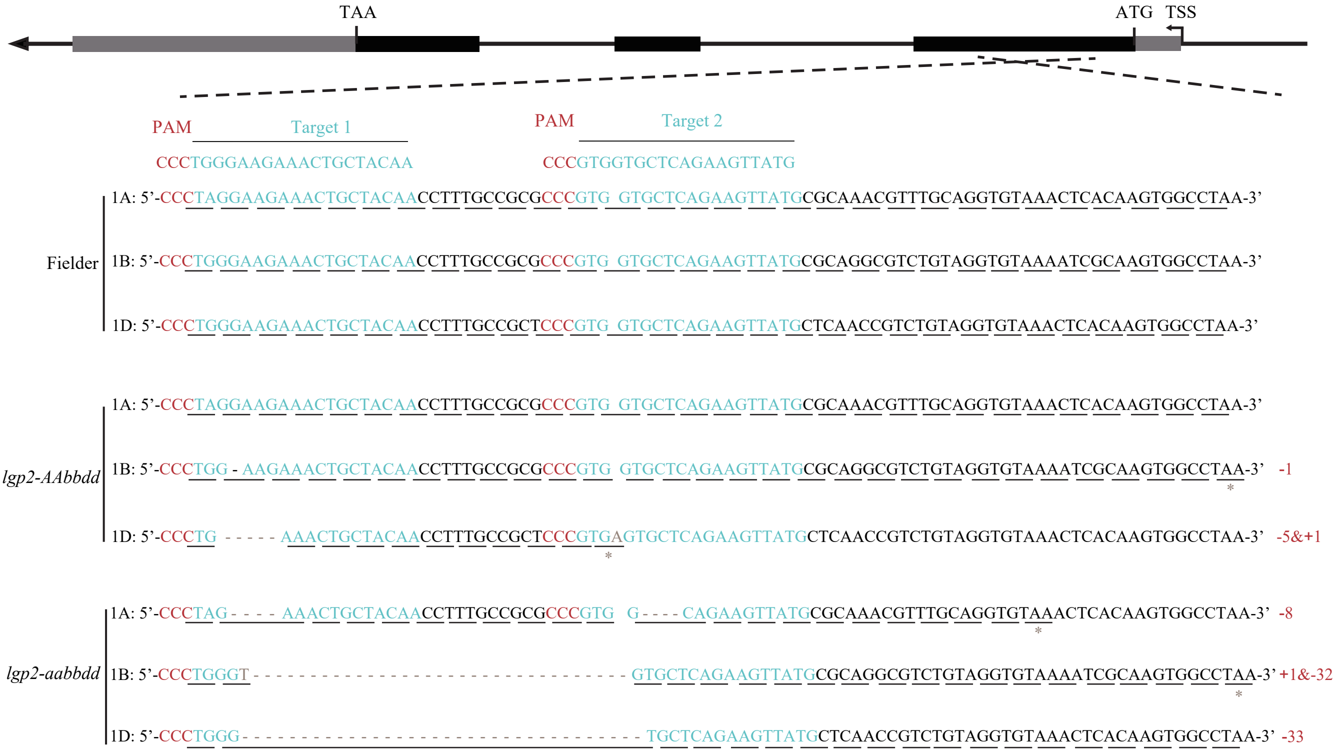

Figure S9. Gene structure of *LGP2*, with targets for CRISPR/Cas9-specific binding to the first exon of this gene, and sequence alignment of *LGP2* in Fielder and the knockdown lines.

Upper panel: Sequence of the sgRNA used to target a region in the first exon encoding the DNA-binding domain conserved among the three *LGP2* homoeologs. Lower panel: Sequences of Fielder (WT) and the two *lgp2* mutants around the sgRNA binding site in the three *LGP2* homoeologs. The protospacer-adjacent motif (PAM) sequence is indicated in red. The target sequence is indicated in light blue. Nucleotide insertions are indicated in brown. "-" indicate deletions. Premature termination codons are denoted with "*". Numbers indicate the number of base pairs of the insertions and deletions. Individual codons in the DNA sequence are underlined. Gray boxes indicate 5'UTR or 3'UTR. Black boxes indicate exons. TSS indicates the transcription start site.

A

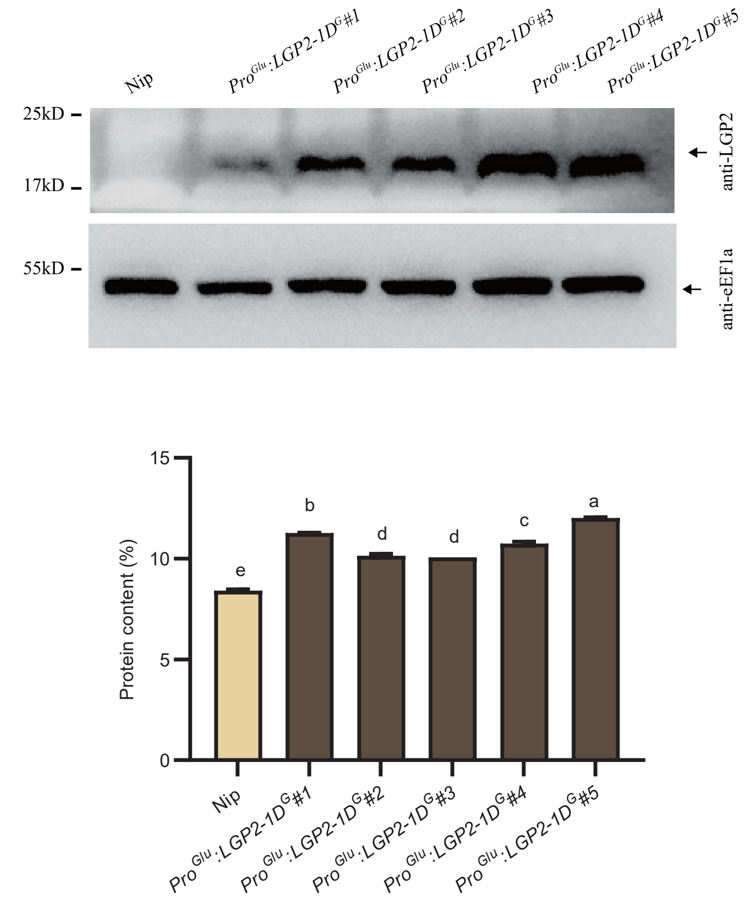

Figure S10. Characterization of transgenic rice lines.

(A) Immunoblot analysis of Nipponbare (Nip; non-transgenic control) and *LGP2*-expressing transgenic rice lines. **(B)** Protein content of Nip and *LGP2*-expressing transgenic rice lines. Data are means ± s.d. (n = 3 biologically independent samples). Group differences were assessed by one-way ANOVA followed by Tukey's Honestly Significant Difference test. Different letters indicate significant differences at $P < 0.05$.. Source data are provided as a Source Data file.

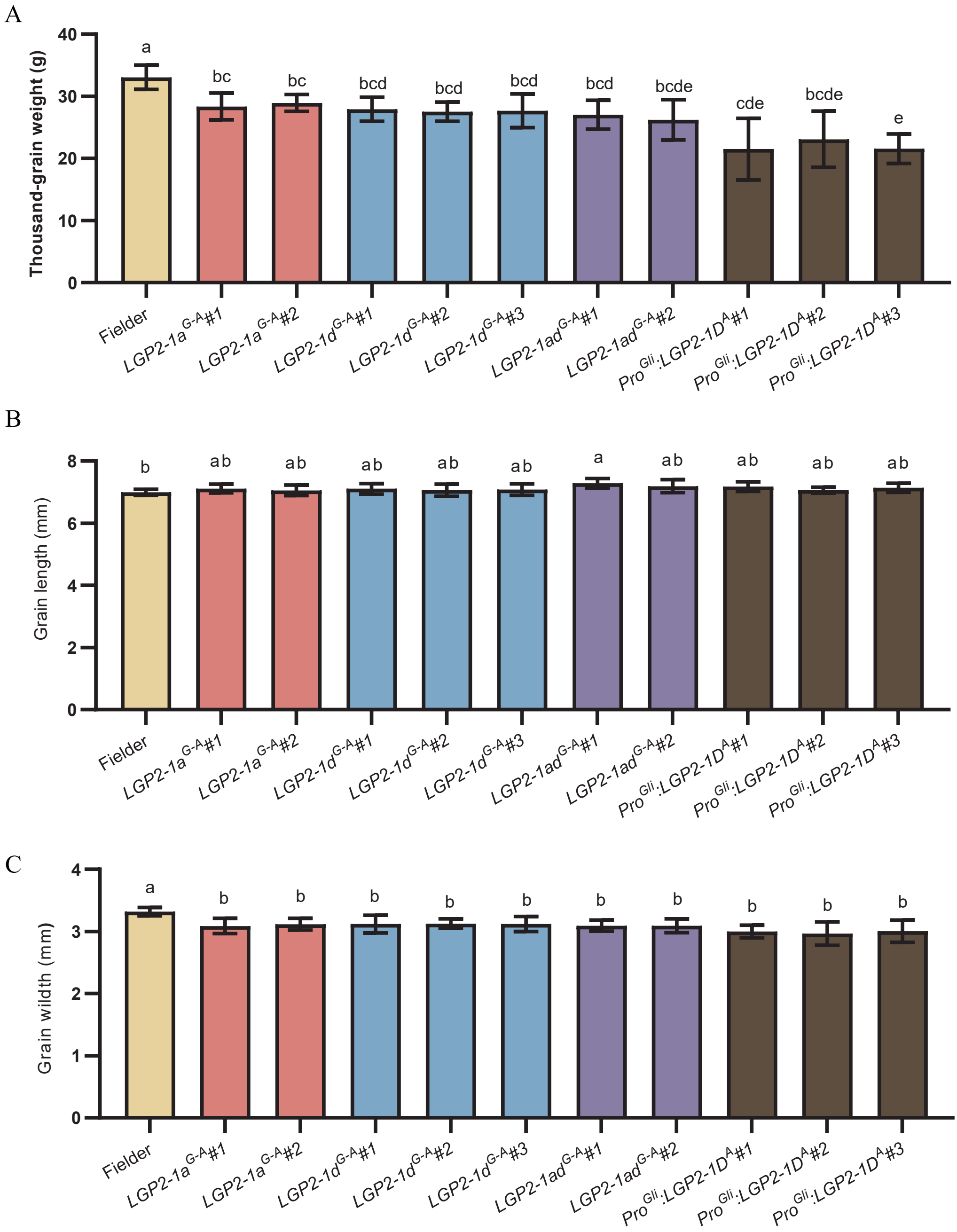

Figure S11. Yield traits of *LGP2* genome-edited lines.

(A–C) Thousand-grain weight **(A)**, grain length **(B)**, and grain width **(C)** of the genome-edited lines *LGP2-1a*$^{G\text{-}A}$, *LGP2-1d*$^{G\text{-}A}$, and *LGP2-1ad*$^{G\text{-}A}$ and transgenic *Pro*^{Gli}*:LGP2-1D*^{A} lines compared with those of Fielder (non-transgenic control). Data are means ± s.d. (n = 10 biologically independent samples). In (A and C), Group differences were assessed by Welch's ANOVA followed by Games-Howell post-hoc tests. In (B), Group differences were assessed by one-way ANOVA followed by Tukey's Honestly Significant Difference test. Different letters indicate significant differences at $P < 0.05$. Source data are provided as a Source Data file.
